# Live imaging and tracking of genome regions in CRISPR/dCas9 knock-in mice

**DOI:** 10.1186/s13059-018-1530-1

**Published:** 2018-11-08

**Authors:** Jinzhi Duan, Guangqing Lu, Yu Hong, Qingtao Hu, Xueying Mai, Jing Guo, Xiaofang Si, Fengchao Wang, Yu Zhang

**Affiliations:** 1 Graduate Program, Peking Union Medical College and Chinese Academy of Medical Sciences, Beijing, 100730 China; 2 Peking University-Tsinghua University-National Institute of Biological Sciences Joint Graduate Program, School of Life Sciences, Peking University, Beijing, 100871 China; 3 National Institute of Biological Sciences, Beijing, 102206 China; 4 Tsinghua Institute of Multidisciplinary Biomedical Research, Tsinghua University, Beijing, 100084 China

**Keywords:** CRISPR/dCas9, dCas9-EGFP, Knock-in mice, Genome labeling, Live imaging, Hepatocytes, Telomere dynamics

## Abstract

CRISPR/dCas9 is a versatile tool that can be used to recruit various effectors and fluorescent molecules to defined genome regions where it can modulate genetic and epigenetic markers, or track the chromatin dynamics in live cells. In vivo applications of CRISPR/dCas9 in animals have been challenged by delivery issues. We generate and characterize a mouse strain with dCas9-EGFP ubiquitously expressed in various tissues. Studying telomere dynamics in these animals reveals surprising results different from those observed in cultured cell lines. The CRISPR/dCas9 knock-in mice provide an important and versatile tool to mechanistically study genome functions in live animals.

**Electronic supplementary material:**

The online version of this article (10.1186/s13059-018-1530-1) contains supplementary material, which is available to authorized users.

## Background

Revolutionary CRISPR/Cas9 technique is becoming one of the most powerful tools in biological and biomedical studies for almost all model organisms [1–4]. While extensive efforts have been focused on the optimization and implication of targeting and cleavage by CRISPR/Cas9 systems for genome editing, recently, the nuclease-deactivated Cas9 (dCas9) has also been developed as a versatile tool to genetically and epigenetically modulate the targeted genomic locus and label the genomic loci in living cells [5–9].

The localization and dynamics of particular genomic locus in 3-dimensional (3D) nuclei have been proposed to regulate various genome functions including gene transcription, DNA recombination, DNA replication, and DNA repair [10–12]. Dissecting the mechanistic roles of chromatin dynamics in physiological in vivo settings will be greatly facilitated by CRISPR genome labeling strategies. However, the dCas9/gRNA tools have been mostly developed in cell culture systems where the dCas9, gRNA, and effector expression cassettes could be transfected or infected into the cells. For in vivo applications of dCas9/gRNA in live animals, how to efficiently deliver all these components, especially the large dCas9 expression cassettes, into the cells of various tissues remains to be a major difficulty [13].

To pave the way for in vivo applications of dCas9/gRNA tools in live animals, we generated mouse strain in which dCas9-EGFP was ubiquitously expressed. The transgenic expression of the dCas9 proteins partially solved the major delivery issue associated with large dCas9 protein. Interestingly, studying telomere dynamics in these animals revealed surprising results different from those observed in cultured cell lines. The CRISPR/dCas9 knock-in mice provide important and versatile tools to precisely study epigenetic and genetic regulations of genome functions in live animals.

## Results and discussion

**Fig. 1:**
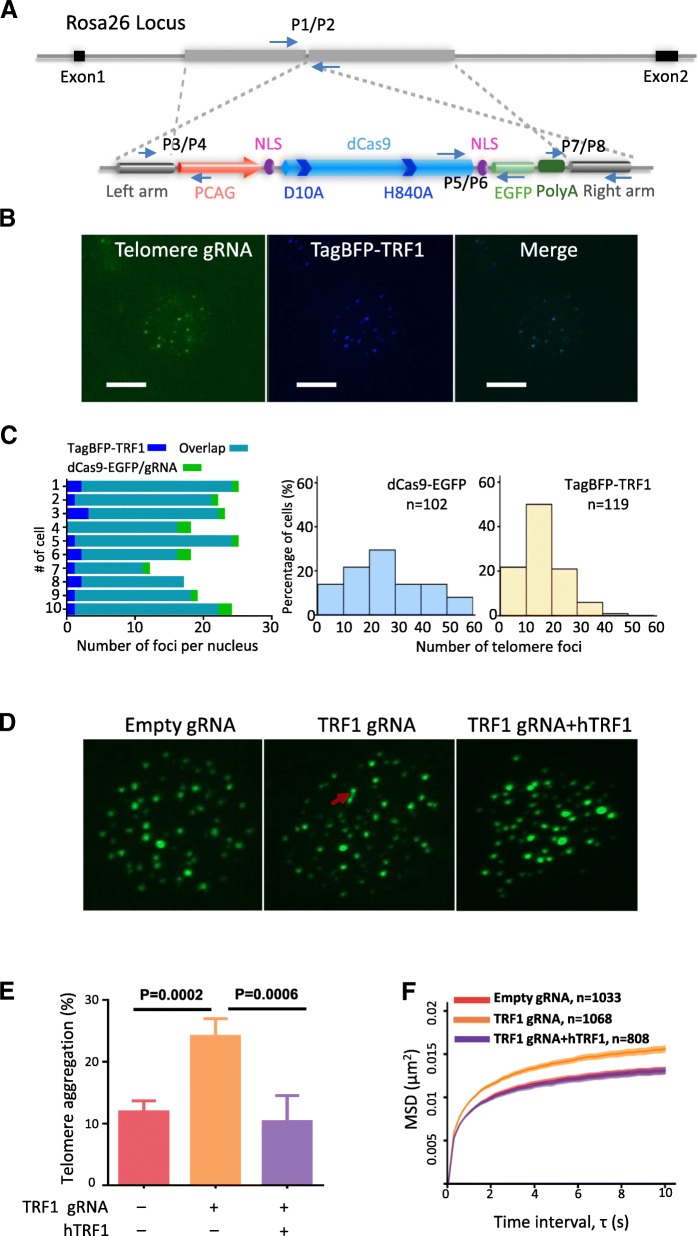
CRISPR imaging of telomeres in dCas9-EGFP knock-in mice. **a** Schematic diagram of dCas9-EGFP Rosa26 targeting vector. **b** Labeling of telomeres in hepatocytes of dCas9-EGFP mice. TagBFP-TRF1 was used as control. **c** Quantification of telomere labeling specificity based on co-localization with TagBFP-TRF1 (left panel). Histograms of telomere foci formation efficiency represented by foci numbers in individual nucleus (right panel). The data were collected from at least two mice for each treatment. **d** Representative images of telomere aggregations observed in dCas9-EGFP mice injected with empty gRNA, gRNAs targeting TRF1, and TRF1 gRNAs plus human TRF1 expression cassette. Aggregation is marked by a red arrow. **e** Quantification of telomere aggregations in dCas9-EGFP mice injected with different constructs. A two-sided *t* test was used for statistical comparison. The data were collected from at least three mice for each treatment. **f** The average MSD curves of telomere in dCas9-EGFP mice injected with different constructs. For the empty gRNA group, 1033 foci were collected in 85 cells from three mice. For TRF1 gRNA group, 1068 foci were collected in 98 cells from four mice. For TRF1 gRNA+TRF1 group, 808 foci were collected in 68 cells from four mice. The data are displayed as mean ± SE

We target-inserted dCas9 (D10A/H840A) expression cassette driven by the ubiquitous CMV early enhancer/chicken β actin (CAG) promoter into the intron 1 of mouse Rosa26 locus (Fig. 1a and Additional file 1: Figure S1A). The cassette expresses a dCas9-EGFP fusion protein, which primarily allows in vivo labeling and dynamic tracking of the gRNA-targeted genomic loci in live animals. In addition, in combination with gRNA-binding effectors [14, 15] or GBP (GFP-binding protein)-effector fusions [16], dCas9-EGFP could also achieve sequence-specific genetic and epigenetic remodulations. By RT-qPCR and western blot analysis (Additional file 1: Figure S1B-C), we verified that the dCas9-EGFP could be widely expressed in various mouse tissues such as brain, heart, kidney, liver, lung, and spleen. Moreover, the expression of dCas9-EGFP could be detected by FACS analysis in all examined hematopoietic cell types including myeloid cells, neutrophils, and B- and T-cells isolated from the bone marrow, spleen, and thymus (Additional file 1: Figure S1D-E). The dCas9-EGFP knock-in mice developed normally, were fertile, and could be bred to homozygosity. We have also verified the gRNA-dependent CRISPR activation and repression functions of the dCas9-EGFP in ex vivo culture for bone marrow-derived dendritic cells (BMDCs) and liver hepatocytes (data not shown).

The dCas9-EGFP knock-in mice could also facilitate the tracking of specific genomic sequences in live animals. We delivered gRNA expression vectors into dCas9-EGFP mice by hydrodynamic injection [17] (Additional file 1: Figure S2A). After the mice were anesthetized, the dynamics of telomeres in hepatocytes of exposured liver lobes could be recorded by a high-speed spinning disk confocal microscope [18] (Additional file 1: Figure S2A and Additional file 2: Movie S1). We found that telomere gRNA could lead to specific and efficient labeling of mouse telomeres, which correlated well with co-injected TagBFP-TRF1 in dCas9-EGFP mouse liver (Fig. 1b, c and Additional file 1: Figure S2B). In addition, major satellite repeats as well as a single genomic locus in X chromosome could also be efficiently labeled in vivo by their specific gRNAs (Additional file 1: Figure S3A-B).

The subunits of telosome/shelterin complex, such as TRF1, TRF2, and TIN2, play important roles in telomere length regulation and end protection [19, 20]. The dCas9-EGFP knock-in mice provide unique tools to dissect the roles of mechanistic factors, such as shelterin factors, in regulating telomere dynamics in live animals. While dCas9-EGFP could be used to live track the dynamics of defined genome regions such as telomeres, by combining with effectors and gRNAs targeting particular genes, the potential roles of those genes could be studied at the same time (Additional file 1: Figure S4A-B). We injected a single transposon vector expressing a standard gRNA-targeting telomeres, Casilio gRNAs targeting TRF1 gene, and PUFc-TagBFP-KRAB into dCas9-EGFP mice. As shown in Fig. 1d, e, inhibition of TRF1 gene transcription in hepatocytes by CRISPRi led to significant telomere aggregation/fusion revealed by CRISPR imaging, which is consistent with previous results obtained in cells and mice conditionally deleted for TRF1 [21, 22]. At the same time, single-particle tracking telomere dynamics demonstrated the increase in microscopic diffusion speed by TRF1 CRISPRi (Fig. 1f). Importantly, all these phenotypes could be completely rescued by overexpression of an exogenous human TRF1 protein (Fig. 1d–f). The dCas9-EGFP knock-in mice could be used to study other mechanistic factors regulating the chromatin dynamics of telomeres and other genome regions in vivo.

**Fig. 2:**
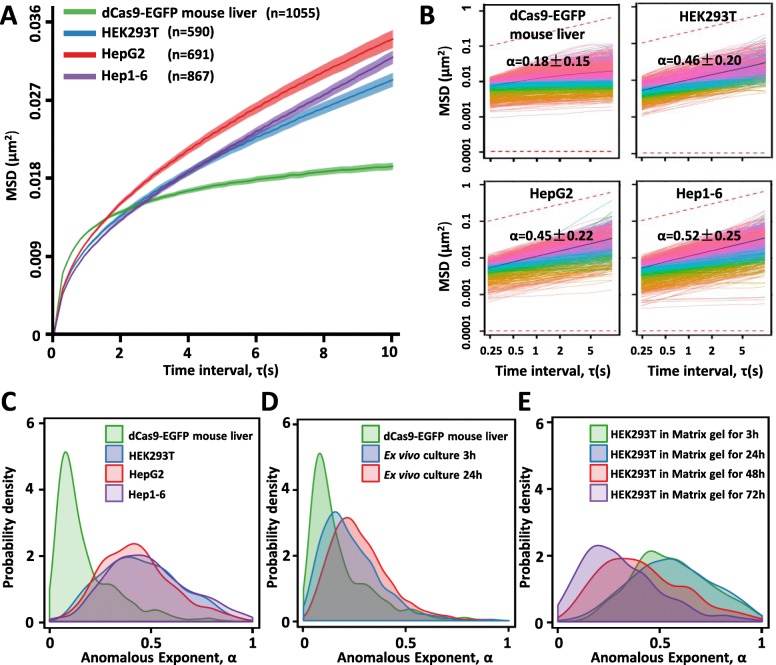
Unique features of telomere dynamics in mouse liver revealed by dCas9-EGFP knock-in mice. **a** The average MSD curves of telomeres in dCas9-EGFP mouse liver and cultured cell lines (HEK293T, HepG2, and Hep1-6). The data are displayed as mean ± SE. **b** MSD curves of individual telomeres (colored curves) and the average MSD curves (bold black curve with shaded area indicating ± SE) as a function of time interval between observations. The upper red dashed line: slope = 0.5. The bottom red dashed line: slope = 0. **c** Distribution of *α* values calculated for individual telomeres of dCas9-EGFP mouse liver and cultured cell lines (HEK293T, HepG2, and Hep1-6). **d** Distribution of *α* values calculated for individual telomeres of dCas9-EGFP mouse liver and ex vivo hepatocytes cultured for 3 and 24 h. **e** Distribution of *α* values calculated for individual telomeres of HEK293T cells cultured in Matrix gel for 3, 24, 48, and 72 h (cells at 3 and 24 h were single cells without cell-cell interactions, and cells at 48 and 72 h were within cell clusters with cell-cell interactions). The data were collected from at least three mice

Interestingly, while the telomeres in hepatocytes within intact liver organ of a live mouse also showed an anomalous subdiffusion as observed in cultured cell lines [23], they demonstrated a mean-squared displacement (MSD) curve very different from culture cell lines such as human HEK293T as well as human HepG2 and mouse Hep1-6 cell lines (Fig. 2a and Additional file 1: Figure S2C). While MSD, <*r*^2^(*τ*)>,was calculated with *τ* designating a time-lag along the trajectory, anomalous subdiffusion could also be described by <*r*^2^(*τ*) > = *D*_*α*_*τ*^*α*^, where *α* < 1 and is an indicator of interactions of the genomic regions with constituents of the nucleoplasm [24]. The telomere dynamics observed in dCas9-EGFP mouse livers showed anomalous diffusion with average *α* as 0.18 (Fig. 2b). However, the telomere diffusion in cultured cell lines such as human HEK293T, human HepG2, and mouse Hep1-6 showed average *α* value near 0.5 (Fig. 2b, c), similar as published in other mammalian cell lines including U2OS, HeLa, NIH3T3, and MEFs [24]. The smaller *α* value indicated the slower and more localized telomere motion in mouse liver cells than that in culture cell lines. We then set up the ex vivo hepatocyte culture from dCas9-EGFP mice which had been hydrodynamically injected with telomere gRNA. Interestingly, *α* value of the anomalous diffusion in those ex vivo cultured cells increased significantly along longer time culture (average *α* was 0.28 after 24 h culture on gelatinized plates) (Fig. 2d and Additional file 1: Figure S4C). On the other hand, after HEK293T and HepG2 cells labeled with dCas9-EGFP and telomere gRNA were cultured in matrix gel, *α* values of telomere diffusion decreased significantly along time (Fig. 2e and Additional file 1: Figure S4D).

Live tracking telomeres in animals opens a new window to mechanistically study telomere functions and regulations in vivo. The telomere dynamics in dCas9-EGFP knock-in mouse liver demonstrated more constrained anomalous diffusion than that observed in cultured cell lines, suggesting that cellular context such as cell-cell interactions and cell-ECM interactions might be involved in regulating the telomere dynamics. It could be speculated that the dynamics of telomere as well as other genomic loci might be tissue-specific and developmentally regulated, which could be studied by the dCas9-EGFP knock-in mice.

## Conclusions

To extend CRISPR/dCas9 tool to live animals, we generated mouse strain with dCas9-EGFP ubiquitously expressed in multiple tissues, which bypasses the delivery issues with large dCas9 protein into animals. We also developed CRISPRimaging-interference (CRISPRii) method [14] to dissect the roles of mechanistic factors in regulating in vivo genome dynamics (Additional file 1: Figure S4A). The telomere dynamics observed in live animals is significantly different from what has been obtained in culture cells. The dCas9-EGFP knock-in mouse strain provides a versatile tool to dissect genome functions and to study chromatin dynamics in live animals.

## Methods

### Oligos and plasmids

A list of gRNAs and primers used in this work is presented in Supplementary information: Additional file 1: Table S1. Schemes and nucleotide sequences for plasmids generated in this work are listed in Supplementary information: Additional file 1: Table S2.

### Experimental animals

The dCas9-EGFP mice were generated by microinjections of a mixture of Cas9 mRNA (80 ng/μl), Rosa26 gRNA (40 ng/μl), and donor fragment (8 ng/μl) into C57BL/6J mouse zygotes. They were genotyped with primer P1–8: P1 and P2 generating 297-bp product to detect wild type allele; P5 and P6 generating 502-bp product to detect dCas9-EGFP knock-in allele; P3 and P4 to detect the left arm; P7 and P8 to detect the right arm (Additional file 1: Figure S1A).

### Cell lines

HEK293T, Hep1-6, and HepG2 cells were cultured in DMEM with 10% FBS, 1% penicillin/streptomycin (GIBCO), and L-glutamine (GIBCO), at 37 °C and 5% CO_2_.

### HEK293T and HepG2 3D culture in Matrigel

Trypsinized HEK293T or HepG2 cells were resuspended in 30 μl complete medium at 5x10^6^ cells/ml and mixed with 270 μl Matrigel matrix solution (5 mg/ml, Corning, 356231). After 1.5 ml complete media was gently added, the culture was kept at 37 °C and 5% CO_2_.

### Hydrodynamic tail vein injection

Plasmids were prepared using the PureYield™ Plasmid Midiprep System (Promega) and resuspended in PBS at a final volume of 10% of the mouse body weight, and injected into tail vein of 6–8-week-old dCas9-EGFP mice within 3–7 s.

### Isolation and ex vivo culture of mouse primary hepatocytes

The liver was perfused during portal vain with 50 ml PTH perfusion solution (160.8 mM NaCl, 3.15 mM KCl, 0.7 mM Na_2_HPO_4_, 33 mM HEPES, pH 7.65) containing 2 mM EDTA (37 °C, 7.5 ml/min), followed by perfusion of 40 ml PTH perfusion solution containing 3 mM CaCl_2_ and 0.5 mg/ml type IV collagenase. Then, the tissue was manually disrupted in PTH solution and passed through a 70-μm nylon filter. The cell pellets were centrifuged and washed by PTH solution twice at 40×*g* for 3 min at 4 °C.

For ex vivo culture, the cells were cultured on 0.1% gelatin-coated cell culture dishes in DMEM with 10% FBS, 1% penicillin/streptomycin, 1% L-glutamine, and 1% Antibiotic-Antimycotic (Thermo Fisher).

For TRF1 repression analysis, after PI staining, GFP-positive and PI-negative hepatocytes were sorted by BD FACSAria II.

### Western blot analysis

Anti-Cas9 mAb (Active motif, Cat#61578) (1:5000) and anti-α-tubulin (Sigma-Aldrich, Cat#T6199) (1:10000) were used as primary antibody. Secondary antibody was IRDye® 800CW Goat anti-Mouse IgG (1:10000, LI-COR, P/N 925–32210). The membranes were scanned on an Odyssey imager (LI-COR).

### Flow cytometry

Single-cell suspensions were prepared from the spleen, bone marrow, and thymus. Red blood cells were lysed by homemade RBC lysis buffer, and remaining cells were incubated with antibodies in PBS for 30 min at 4 °C and washed with PBS twice before analyzed by BD FACSAria III. Antibodies used for staining include as follows: anti-B220-PE (eBiosciences, 12-0452-83), anti-TCRβ-APC (eBiosciences, 17-5961-82), anti-CD4-PE (eBiosciences, 12-0041-85), and anti-MHCII-APC (eBiosciences, 17-5321-81). FACS results were analyzed by FlowJo.

### RNA extraction, RT-qPCR

RNA was extracted using the Direct-zol™ RNA MiniPrep Kit (Zymo Research, Cat#R2051). cDNA was synthesized by the ImProm-II™ Reverse Transcriptase system (Promega, Cat#A3801) using 100 ng of RNA per reaction. The qPCR reactions were prepared with the KAPA SYBR® FAST qPCR Kit (KAPA, Cat#KK4601) using 1 μl of cDNA per reaction in a 20 μl total reaction volume. The relative gene expression levels were normalized to GAPDH.

### Live animal imaging

For dCas9-EGFP mice, 25 μg Telo-gRNA vector was injected. For colocalization experiments, 3 μg CMV-TagBFP-TRF1 was also injected. Forty-eight hours after injection, mice were anesthetized and liver lobes were exposed and imaged as previously described [18]. All images (512 × 512 pixels) were acquired by a spinning disk confocal microscope (PerkinElmer) with 100× oil immersion objective, which is equipped with four different lasers (excitation at 405, 488, 561, and 633 nm) and emission band-pass filters at 450/50 (channel 1), 515/30 (channel 2), 590/50 nm (channel 3), and 670/50 nm (channel 4). For dynamic telomere tracking, a fixed layer of the corresponding channel was acquired by four frames every second for a total of 120 s. For the telomere co-localization, a z-stack of multiple layers which covered the whole cells with a step size of 0.5 μm was acquired.

For TRF1 repression, 30 μg Ppb-multi-gTRF1-CAG-Pufc-TagBFP-KRAB and 30 μg pCMV-hyPBase were delivered into dCas9-EGFP mouse liver by hydrodynamic tail vein injection. Seven days later, the mouse liver telomere imaging was taken following the instruction above.

### Imaging data analysis

Z-stack images were taken with a step size of 500 nm and enough steps to cover the depth of each nucleus. Foci number counting was performed by the “Measurement, Find Objects” function in Volocity software. Telomere movies were analyzed by MATLAB tracking package “u-track” [25]. Only cells with ≥ 5 foci were kept for further analyses. Nucleus drift correction was performed by subtracting the movement of cell center from each trajectory. Trajectories of each foci were drawn by linking identified puncta to their nearest neighbors within a maximum distance range of three pixels (198.9 nm) in the previous frame using custom scripts. Trajectories which lost more than half of total number of frames were discarded.

For each trajectory, the MSD as a function of time delay *t* = *n*Δ*t* was calculated by $\mathrm{MSD}\left(n\Delta t\right)=\frac{1}{N-1-n}\sum \limits_{i=1}^{N-1-n}{\left|r\left(i\Delta t+n\Delta t\right)-r\Big(i\Delta t\left.\Big)\right|\right.}^2$ where Δ*t* is the frame length (0.25 s for dCas9-EGFP), *n* is the number of frames in a time delay, *N* is the total number of frames, and *r*(*t*) is the two-dimensional coordinate. The consecutive analysis of MSD curves was carried out using MATLAB package “@msdanalyzer” [26]. The MSD curves were fitted by least-squares regression to a model for confined diffusion and macroscopic diffusion by [27],

$$ \mathrm{MSD}(t)=A\left(1-{e}^{-t/\widehat{o}}\right)+4{D}_{\mathrm{macro}}t $$

where *A* is the confinement area, *τ* is a constant from which the microscopic diffusion coefficient *D*_micro_ = *A*/4*τ* can be derived, and *D*_macro_ is the macroscopic diffusion coefficient. The confinement size *L* was calculated by $L=\sqrt{A/2}$.

To study the anomalous diffusion, MSD curve for each trajectory was fitted by least-squares regression to the general motion equation [24]:

$$ \mathrm{MSD}(t)=4{D}_{\alpha }{t}^{\alpha } $$

Telomere aggregations were counted per nucleus, defined as at least two telomeres clustering together with a maximum spot-to-spot distance of half of a telomere spot diameter based on their 3D reconstruction [21].

### Quantification and statistical analysis

All results were presented as the mean ± SD and *p* values of < 0.05 or below were considered significant.

## Additional files

Additional file 1: **Figure S1.** Characterization of dCas9-EGFP knock-in mice. **Figure S2.** Visualizing telomere dynamics in dCas9-EGFP knock-in mouse liver. **Figure S3.** CRISPR in vivo imaging of major satellite and single genomic locus in X chromosome in dCas9-EGFP knock-in mice. **Figure S4.** Compare the telomere dynamics in vivo and in vitro. **Table S1.** Primer sequences. **Table S2.** Sequences of plasmids. (PDF 1413 kb)

Additional file 2: **Movie S1.** dCas9-EGFP mouse liver telomere tracking. (MOV 9593 kb)
