# Supplementary material for: Live imaging and tracking of genome regions in CRISPR/dCas9 knock-in mice
Source: Genome Biol. 2018 Nov 8;19:192. doi: 10.1186/s13059-018-1530-1 (PMC6225728; doi:10.1186/s13059-018-1530-1)
Supplement: Supplementary file 1 — Figure S1. Characterization of dCas9-EGFP knock-in mice. Figure S2. Visualizing telomere dynamics in dCas9-EGFP knock-in mouse liver. Figure S3. CRISPR in vivo imaging of major satellite and single genomic locus in X chromosome in dCas9-EGFP knock-in mice. Figure S4. Compare the telomere dynamics in vivo and in vitro. Table S1. Primer sequences. Table S2. Sequences of plasmids. (PDF 1413 kb) [file 13059_2018_1530_MOESM1_ESM.pdf]

Supplemental Information

Figure S1. Characterization of dCas9-EGFP knock-in mice.

Figure S2. Visualizing telomere dynamics in dCas9-EGFP knock-in mouse liver.

Figure S3. CRISPR in *vivo* imaging of major satellite and single genomic locus in X chromosome in dCas9-EGFP knock-in mice.

Figure S4. Compare the telomere dynamics *in vivo* and *in vitro*.

Table S1: Primer sequences

Table S2: Sequences of plasmids

Figure S1. Characterization of dCas9-EGFP knock-in mice.

(A) Genotyping results of the dCas9-EGFP knock-in mouse strain. (B) RNA expression analysis of dCas9 in different dCas9-EGFP mouse tissues by RT-qPCR. The data are presented as relative expression level after normalization with GAPDH. (C) The Western blotting analysis of dCas9-EGFP protein expression in different mouse tissues. The α-tubulin was used as a loading control. (D) FACS gating strategies for mouse bone marrow, spleen, and thymus. (E) FACS detection of GFP fluorescence in different immune cells from dCas9-EGFP mice. The data were obtained from at least two mice for each experiment.

Figure S2. Visualizing telomere dynamics in dCas9 knock-in mouse liver.

(A) The schematic of *in vivo* CRISPR imaging of telomeres in dCas9-EGFP mouse livers. (B) Representative images for control gRNA and without gRNA in dCas9-EGFP mouse liver. (C) Representative trajectories of telomeres in one hepatocyte of dCas9-EGFP mouse liver (scale bar, 5 μm) and trajectories of three individual telomeres with different confined regions (scale bars, 200 nm). The trajectory length is 474 frames. See also Movie S1. The data were obtained from at least two mice for each experiment.

Figure S3. CRISPR *in vivo* imaging of major satellites and a single genomic locus in X chromosome in dCas9-EGFP knock-in mice.

(A) Labeling of major satellite in hepatocytes of dCas9-EGFP mice. TagBFP-TRF1 was used as control. Histograms of major satellite foci number distribution in individual nucleus (lower panel). (B) Labeling of a single genomic locus in X chromosome in

dCas9-EGFP mice. Representative images for labeling in male and female mice (left panel). Histograms of foci number distribution in individual nucleus of male and female mice (łright panel). The data were obtained from at least two mice for each experiment.

Figure S4. Compare the telomere dynamics *in vivo* and *in vitro*.

(A) The schematic of CRISPR imaging and interference (CRISPRii) strategy in dCas9-EGFP mouse. (B) RT-qPCR analysis of TRF1 repression by CRISPRi. The data are presented as relative expression fold change comparing TRF1 gRNA and control gRNA injected dCas9-EGFP mice. (C) MSD curves of individual telomeres (colored curves) and the average MSD curves (bold black curve with shaded area indicating ± SE) as a function of time interval between observations. The upper red dashed line: slope=0.5. The bottom red dashed line: slope=0. (D) The left panel is MSD curves of individual telomeres (colored curves) and the average MSD curves (bold black curve with shaded area indicating ± SE) as a function of time interval between observations for HepG2 cells in matrix gel cultured for 72 hours. The upper red dashed line: slope=0.5. The bottom red dashed line: slope=0. The right panel is distribution of α values calculated for individual telomeres of HepG2 cultured on plate (blue) and in Matrix gel for 72h (green). (E) Summary of MSD analysis (Data are shown as mean ± SD)

Table S1: Primer sequences

Name	Sequence	Note
Rosa-SG	GGCATTCTACACGTTATTGCTGG	Knock-in site
TRF1-re-SG1	CACGGCGCCAGCTGAGGCA	Repression site
TRF1-re-SG2	GAAGGCTCGGCACAGAGAC	Repression site
TRF1-re-SG3	GGGACGCGCCGAGCCGTGA	Repression site
Telo-sg	GTTAGGGTTAGGGTTAGGGTTA	Labeling site, telomeres
Majset-sg	CCATATTCCACGTCCTACAG	Labeling site, major satellites
X386-sg	CACACACAGCAGAGGATGTG	Labeling site, X chromosome
P1	GTACACATCTGTAAAAGGTGGTTCC	Genotyping primer
P2	TAGAGCACAAGCACACACAACAC	Genotyping primer
P3	CCATAGAAAAGCCTTGACTTGAGGTTAG	Genotyping primer
P4	ATGGGCTATGAACTAATGACCCCG	Genotyping primer
P5	TTACGAGAAGCTGAAGGGG	Genotyping primer/dCas9 qPCR primer
P6	GAACTTGTGGCCGTTTACGT	Genotyping primer/dCas9 qPCR primer
P7	AAGAGAATAGCAGGCATGCTGG	Genotyping primer
P8	GACTTTGGCTGTGAAGAATTTGGAT	Genotyping primer
TRF1-F	TTTCGTCGTACTCGTGACAGCG	qPCR primer
TRF1-R	GGGAGTGGAACACATCCTTCTGA	qPCR primer
GAPDH-F	TGAAGCAGGCATCTGAGGG	qPCR primer
GAPDH-R	CGAAGGTGGAAGAGTGGGAG	qPCR primer

Table S2: Sequences of plasmids

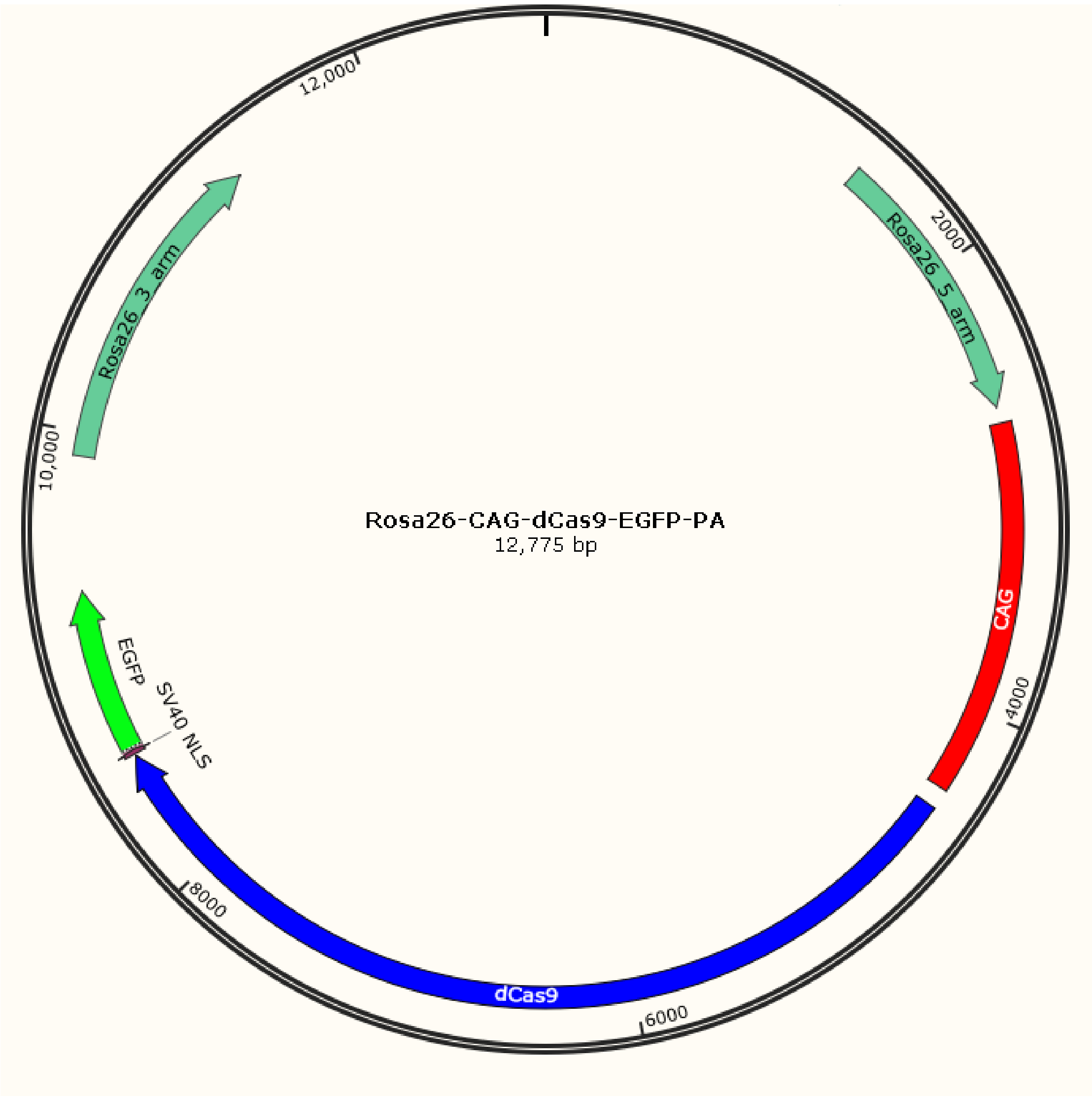

Rosa26-CAG-dCas9-EGFP-PA

tggtcctgcaactttatccgcctccatccagtctattaattgttgccgggaagctagagtaagtagttcgccagttaatagtttgcgcaacgttgttgccattgctac
aggcatcgtggtgtcacgctcgtcgtttggtatggcttcattcagctccggttcccaacgatcaaggcgagttacatgatcccccatgttgtgcaaaaaagcggtta
gctccttcggtcctccgatcgttgtcagaagtaagttggccgcagtgttatcactcatggttatggcagcactgcataattctcttactgtcatgccatccgtaagat
gcttttctgtgactggtgagtactcaaccaagtcattctgagaatagtgtatgcggcgaccgagttgctcttgcccggcgtcaatacgggataataccgcgccacat
agcagaactttaaaagtgctcatcattggaaaacgttcttcggggcgaaaactctcaaggatcttaccgctgttgagatccagttcgatgtaacccactcgtgcac
ccaactgatcttcagcatcttttactttcaccagcgtttctgggtgagcaaaaacaggaaggcaaaatgccgcaaaaaagggaataagggcgacacggaaatgt
tgaatactcatactcttcctttttcaatattattgaagcatttatcagggttattgtctcatgagcggatacatatttgaatgtatttagaaaaataaacaaataggg
gttccgcgcacatttccccgaaaagtgccacctaaattgtaagcgttaatattttgttaaaattcgcgttaaatttttgttaaatcagctcattttttaaccaataggc
cgaaatcggcaaaatcccttataaatcaaaagaatagaccgagatagggttgagtgttgttccagtttggaacaagagtccactattaaagaacgtggactcca
acgtcaaagggcgaaaaaccgtctatcagggcgatggcccactacgtgaaccatcaccctaatcaagttttttggggtcgaggtgccgtaaagcactaaatcgg
aaccctaaagggagcccccgatttagagcttgacggggaaagccggcgaacgtggcgagaaaggaagggaagaaagcgaaaggagcgggcgctagggcgc
tggcaagtgtagcggtcacgctgcgcgtaaccaccacacccgccgcgcttaatgcgccgctacagggcgcgtcccattcgccattcaggctgcgcaactgttggg
aagggcgatcggtgcgggcctcttcgctattacgccagctggcgaaagggggatgtgctgcaaggcgattaagttgggtaacgccagggttttcccagtcacgac
gttgtaaaacgacggccagtgagcgcgcgtaatacgactcactatagggcgaattgggtaccgatatcaggcctgtgcacctcctggcttctgaggaccgccctg

ggcctgggagaatcccttccccctcttccctcgtgatctgcaactccagtctttctagaagatgggcgggagtcttctgggcaggcttaaaggctaacctggtgtgt
gggcgttgtcctgcaggggaattgaacaggtgtaaaattggagggacaagacttcccacagattttcggttttgtcgggaagtttttaataggggcaaataagg
aaaatgggaggataggtagtcatctggggttttatgcagcaaaactacaggttattattgcttgtgatccgcctcggagtattttccatcgaggtagattaaagac
atgctcacccgagttttatactctcctgcttgagatccttactacagtatgaaattacagtgtcgcgagttagactatgtaagcagaattttaatcatttttaaagag
cccagtacttcatatccatttctcccgctccttctgcagccttatcaaaaggtattttagaacactcattttagccccattttcatttattatactggcttatccaacccc
tagacagagcattggcattttccctttcctgatcttagaagtctgatgactcatgaaaccagacagattagttacatacaccacaaatcgaggctgtagctggggc
ctcaacactgcagttcttttataactccttagtacactttttgttgatcctttgccttgatccttaattttcagtgtctatcacctctcccgtcaggtggtgttccacattt
gggcctattctcagtccagggagttttacaacaatagatgtattgagaatccaacctaaagcttaactttccactcccatgaatgcctctctcctttttctccatttat
aaactgagctattaaccattaatggtttccaggtggatgtctcctcccccaatattacctgatgtatcttacatattgccaggctgatattttaagacattaaaaggt
atatttcattattgagccacatggtattgattactgcttactaaaattttgtcattgtacacatctgtaaaaggtggttccttttggaatgcaaagttcaggtgtttgt
tgtctttcctgacctaaggtcttgtgagcttgtatttttttctatttaagcagtgctttctcttggactggcttgactcatggcattctacacgttaattaagctagttatt
aatagtaatcaattacggggtcattagttcatagcccatatatggagttccgcgttacataacttacggtaaatggcccgcctggctgaccgcccaacgacccccg
cccattgacgtcaataatgacgtatgttcccatagtaacgccaatagggactttccattgacgtcaatgggtggactatttacggtaaactgcccacttggcagta
catcaagtgtatcatatgccaagtacgccccctattgacgtcaatgacggtaaatggcccgcctggcattatgcccagtacatgaccttatgggactttcctacttg
gcagtacatctacgtattagtcatcgctattaccatgggtcgaggtgagccccacgttctgcttcactctccccatctcccccccctccccacccccaattttgtattta
tttatttttaattattttgtgcagcgatgggggcgggggggggggggggcgcgcgccaggcggggcggggcggggcgaggggcggggcggggcgaggcggaga
ggtgcggcggcagccaatcagagcggcgcgctccgaaagtttccttttatggcgaggcggcggcggcggcggccctataaaaagcgaagcgcgcggcgggcgg
gagtcgctgcgttgccttcgccccgtgccccgctccgcgccgcctcgcgccgcccgccccggctctgactgaccgcgttactcccacaggtgagcgggcgggacgg
cccttctcctccgggctgtaattagcgcttggtttaatgacggctcgtttcttttctgtggctgcgtgaaagccttaaagggctccgggagggccctttgtgcggggg
ggagcggctcggggggtgcgtgcgtgtgtgtgtgcgtggggagcgccgcgtgcggcccgcgctgcccggcggctgtgagcgctgcgggcgcggcgcggggctttg
tgcgctccgcgtgtgcgcgaggggagcgcggccgggggcggtgccccgcggtgcggggggggctgcgaggggaacaaaggctgcgtgcggggtgtgtgcgtggg
ggggtgagcagggggtgtgggcgcggcggtcgggctgtaacccccccctgcacccccctccccgagttgctgagcacggcccggcttcgggtgcggggctccgtg
cggggcgtggcgcggggctcgccgtgccgggcggggggtggcggcaggtgggggtgccgggcggggcggggccgcctcgggccggggagggctcgggggagg
ggcgcggcggccccggagcgccggcggctgtcgaggcgcggcgagccgcagccattgccttttatggtaatcgtgcgagagggcgcagggacttcctttgtccca
aatctggcggagccgaaatctgggaggcgccgccgcaccccctctagcgggcgcgggcgaagcggtgcggcgccggcaggaaggaaatgggcggggagggcc
ttcgtgcgtcgccgcgccgccgtccccttctccatctccagcctcggggctgccgcagggggacggctgccttcgggggggacggggcagggcggggttcggcttc
tggcgtgtgaccggcggctctagagcctctgctaaccatgttcatgccttcttctttttcctacagctcctgggcaacgtgctggttattgtgctgtctcatcattttgg
caaagaattgattaattcgagcgaacgcgtATATCAAGCTTGCGGTACCGCGGGCCCGGGGgCCACCATGGCTAGCCCCAAAAAGAA
GAGGAAAGTGGACAAGAAGTATTCTATCGGACTGGCCATCGGGACTAATAGCGTCGGGTGGGCCGTGATCACTGACGAG
TACAAGGTGCCCTCTAAGAAGTTCAAGGTGCTCGGGAACACCGACCGGCATTCCATCAAGAAAAATCTGATCGGAGCTCT
CCTCTTTGATTCAGGGGAGACCGCTGAAGCAACCCGCCTCAAGCGGACTGCTAGACGGCGGTACACCAGGAGGAAGAAC
CGGATTTGTTACCTTCAAGAGATATTCTCCAACGAAATGGCAAAGGTCGACGACAGCTTCTTCCATAGGCTGGAAGAATCA
TTCCTCGTGGAAGAGGATAAGAAGCATGAACGGCATCCCATCTTCGGTAATATCGTCGACGAGGTGGCCTATCACGAGAA
ATACCCAACCATCTACCATCTTCGCAAAAAGCTGGTGGACTCAACCGACAAGGCAGACCTCCGGCTTATCTACCTGGCCCT
GGCCCACATGATCAAGTTCAGAGGCCACTTCCTGATCGAGGGCGACCTCAATCCTGACAATAGCGATGTGGATAAACTGTT
CATCCAGCTGGTGCAGACTTACAACCAGCTCTTTGAAGAGAACCCCATCAATGCAAGCGGAGTCGATGCCAAGGCCATTCT
GTCAGCCCGGCTGTCAAAGAGCCGCAGACTTGAGAATCTTATCGCTCAGCTGCCGGGTGAAAAGAAAAATGGACTGTTCG
GGAACCTGATTGCTCTTTCACTTGGGCTGACTCCCAATTTCAAGTCTAATTTCGACCTGGCAGAGGATGCCAAGCTGCAACT
GTCCAAGGACACCTATGATGACGATCTCGACAACCTCCTGGCCCAGATCGGTGACCAATACGCCGACCTTTTCCTTGCTGCT
AAGAATCTTTCTGACGCCATCCTGCTGTCTGACATTCTCCGCGTGAACACTGAAATCACCAAGGCCCCTCTTTCAGCTTCAAT
GATTAAGCGGTATGATGAGCACCACCAGGACCTGACCCTGCTTAAGGCACTCGTCCGGCAGCAGCTTCCGGAGAAGTACA
AGGAAATCTTCTTTGACCAGTCAAAGAATGGATACGCCGGCTACATCGACGGAGGTGCCTCCCAAGAGGAATTTTATAAG
TTTATCAAACCTATCCTTGAGAAGATGGACGGCACCGAAGAGCTCCTCGTGAAACTGAATCGGGAGGATCTGCTGCGGAA
GCAGCGCACTTTCGACAATGGGAGCATTCCCCACCAGATCCATCTTGGGGAGCTTCACGCCATCCTTCGGCGCCAAGAGGA

CTTCTACCCCTTTCTTAAGGACAACAGGGAGAAGATTGAGAAAATTCTCACTTTCCGCATCCCCTACTACGTGGGACCCCTC
GCCAGAGGAAATAGCCGGTTTGCTTGGATGACCAGAAAGTCAGAAGAAACTATCACTCCCTGGAACTTCGAAGAGGTGG
TGGACAAGGGAGCCAGCGCTCAGTCATTCATCGAACGGATGACTAACTTCGATAAGAACCTCCCCAATGAGAAGGTCCTG
CCGAAACATTCCCTGCTCTACGAGTACTTTACCGTGTACAACGAGCTGACCAAGGTGAAATATGTCACCGAAGGGATGAG
GAAGCCCGCATTCCTGTCAGGCGAACAAAAGAAGGCAATTGTGGACCTTCTGTTCAAGACCAATAGAAAGGTGACCGTGA
AGCAGCTGAAGGAGGACTATTTCAAGAAAATTGAATGCTTCGACTCTGTGGAGATTAGCGGGGTCGAAGATCGGTTCAAC
GCAAGCCTGGGTACCTACCATGATCTGCTTAAGATCATCAAGGACAAGGATTTTCTGGACAATGAGGAGAACGAGGACAT
CCTTGAGGACATTGTCCTGACTCTCACTCTGTTCGAGGACCGGGAAATGATCGAGGAGAGGCTTAAGACCTACGCCCATCT
GTTCGACGATAAAGTGATGAAGCAACTTAAACGGAGAAGATATACCGGATGGGGACGCCTTAGCCGCAAACTCATCAAC
GGAATCCGGGACAAACAGAGCGGAAAGACCATTCTTGATTTCCTTAAGAGCGACGGATTCGCTAATCGCAACTTCATGCA
ACTTATCCATGATGATTCCCTGACCTTTAAGGAGGACATCCAGAAGGCCCAAGTGTCTGGACAAGGTGACTCACTGCACGA
GCATATCGCAAATCTGGCTGGTTCACCCGCTATTAAGAAGGGTATTCTCCAGACCGTGAAAGTCGTGGACGAGCTGGTCA
AGGTGATGGGTCGCCATAAACCAGAGAACATTGTCATCGAGATGGCCAGGGAAAACCAGACTACCCAGAAGGGACAGA
AGAACAGCAGGGAGCGGATGAAAAGAATTGAGGAAGGGATTAAGGAGCTCGGGTCACAGATCCTTAAAGAGCACCCGG
TGGAAAACACCCAGCTTCAGAATGAGAAGCTCTATCTGTACTACCTTCAAAATGGACGCGATATGTATGTGGACCAAGAG
CTTGATATCAACAGGCTCTCAGACTACGACGTGGACGCCATCGTCCCTCAGAGCTTCCTCAAAGACGACTCAATTGACAAT
AAGGTGCTGACTCGCTCAGACAAGAACCGGGGAAAGTCAGATAACGTGCCCTCAGAGGAAGTCGTGAAAAAGATGAAG
AACTATTGGCGCCAGCTTCTGAACGCAAAGCTGATCACTCAGCGGAAGTTCGACAATCTCACTAAGGCTGAGAGGGGCGG
ACTGAGCGAACTGGACAAAGCAGGATTCATTAAACGGCAACTTGTGGAGACTCGGCAGATTACTAAACATGTCGCCCAAA
TCCTTGACTCACGCATGAATACCAAGTACGACGAAAACGACAAACTTATCCGCGAGGTGAAGGTGATTACCCTGAAGTCC
AAGCTGGTCAGCGATTTCAGAAAGGACTTTCAATTCTACAAAGTGCGGGAGATCAATAACTATCATCATGCTCATGACGCA
TATCTGAATGCCGTGGTGGGAACCGCCCTGATCAAGAAGTACCCAAAGCTGGAAAGCGAGTTCGTGTACGGAGACTACA
AGGTCTACGACGTGCGCAAGATGATTGCCAAATCTGAGCAGGAGATCGGAAAGGCCACCGCAAAGTACTTCTTCTACAGC
AACATCATGAATTTCTTCAAGACCGAAATCACCCTTGCAAACGGTGAGATCCGGAAGAGGCCGCTCATCGAGACTAATGG
GGAGACTGGCGAAATCGTGTGGGACAAGGGCAGAGATTTCGCTACCGTGCGCAAAGTGCTTTCTATGCCTCAAGTGAACA
TCGTGAAGAAAACCGAGGTGCAAACCGGAGGCTTTTCTAAGGAATCAATCCTCCCCAAGCGCAACTCCGACAAGCTCATT
GCAAGGAAGAAGGATTGGGACCCTAAGAAGTACGGCGGATTCGATTCACCAACTGTGGCTTATTCTGTCCTGGTCGTGGC
TAAGGTGGAAAAAGGAAAGTCTAAGAAGCTCAAGAGCGTGAAGGAACTGCTGGGTATCACCATTATGGAGCGCAGCTCC
TTCGAGAAGAACCCAATTGACTTTCTCGAAGCCAAAGGTTACAAGGAAGTCAAGAAGGACCTTATCATCAAGCTCCCAAA
GTATAGCCTGTTCGAACTGGAGAATGGGCGGAAGCGGATGCTCGCCTCCGCTGGCGAACTTCAGAAGGGTAATGAGCTG
GCTCTCCCCTCCAAGTACGTGAATTTCCTCTACCTTGCAAGCCATTACGAGAAGCTGAAGGGGAGCCCCGAGGACAACGAG
CAAAAGCAACTGTTTGTGGAGCAGCATAAGCATTATCTGGACGAGATCATTGAGCAGATTTCCGAGTTTTCTAAACGCGTC
ATTCTCGCTGATGCCAACCTCGATAAAGTCCTTAGCGCATACAATAAGCACAGAGACAAACCAATTCGGGAGCAGGCTGA
GAATATCATCCACCTGTTCACCCTCACCAATCTTGGTGCCCCTGCCGCATTCAAGTACTTCGACACCACCATCGACCGGAAA
CGCTATACCTCCACCAAAGAAGTGCTGGACGCCACCCTCATCCACCAGAGCATCACCGGACTTTACGAAACTCGGATTGAC
CTCTCACAGCTCGGAGGGGATGAGGGAGCTCCCAAGAAAAAGCGCAAGGTAGGTAGTTCCgtgagcaagggcgaggagctgttc
accggggtggtgcccatcctggtcgagctggacggcgacgtaaacggccacaagttcagcgtgtccggcgagggcgagggcgatgccacctacggcaagctga
ccctgaagttcatctgcaccaccggcaagctgcccgtgccctggcccaccctcgtgaccaccctgacctacggcgtgcagtgcttcagccgctaccccgaccacat
gaagcagcacgacttcttcaagtccgccatgcccgaaggctacgtccaggagcgcaccatcttcttcaaggacgacggcaactacaagacccgcgccgaggtga
agttcgagggcgacaccctggtgaaccgcatcgagctgaagggcatcgacttcaaggaggacggcaacatcctggggcacaagctggagtacaactacaacag
ccacaacgtctatatcatggccgacaagcagaagaacggcatcaaggtgaacttcaagatccgccacaacatcgaggacggcagcgtgcagctcgccgaccac
taccagcagaacacccccatcggcgacggccccgtgctgctgcccgacaaccactacctgagcacccagtccgccctgagcaaagaccccaacgagaagcgcg
atcacatggtcctgctggagttcgtgaccgccgccgggatcactctcggcatggacgagctgtacaagtaaGCGGCCGCGACTCTAGAGTCGACCTG
CAGGCATGCAAGCTTactagtaactcctcaggtgcaggctgcctatcagaaggtggtggctggtgtggccaatgccctggctcacaaataccactgagatc

tttttccctctgccaaaaattatggggacatcatgaagccccttgagcatctgacttctggctaataaaggaaatttattttcattgcaatagtgtgttggaatttttt
gtgtctctcactcggaaggacatatgggagggcaaatcatttaaaacatcagaatgagtatttggtttagagtttggcaacatatgcccatatgctggctgccatg
aacaaaggttggctataaagaggtcatcagtatatgaaacagccccctgctgtccattccttattccatagaaaagccttgacttgaggttagattttttttatattt
tgttttgtgttattttttttctttaacatccctaaaattttccttacatgttttactagccagatttttcctcctctcctgactactcccagtcatagctgtccctcttctctta
tgaagatccctcgacggcgcgcctggtctaaatgtgattttgccaagcttcttcaggacctataattttgcttgacttgtagccaaacacaagtaaaatgattaagc
aacaaatgtatttgtgaagcttggtttttaggttgttgtgttgtgtgtgcttgtgctctataataatactatccaggggctggagaggtggctcggagttcaagagca
cagactgctcttccagaagtcctgagttcaattcccagcaaccacatggtggctcacaaccatctgtaatgggatctgatgccctcttctggtgtgtctgaagacca
caagtgtattcacattaaataaataaatcctccttcttcttcttttttttttttttaaagagaatactgtctccagtagaatttactgaagtaatgaaatactttgtgtt
tgttccaatatggtagccaataatcaaattactctttaagcactggaaatgttaccaaggaactaatttttatttgaagtgtaactgtggacagaggagccataac
tgcagacttgtgggatacagaagaccaatgcagactttaatgtcttttctcttacactaagcaataaagaaataaaaattgaacttctagtatcctatttgtttaaa
ctgctagctttacttaacttttgtgcttcatctatacaaagctgaaagctaagtctgcagccattactaaacatgaaagcaagtaatgataattttggatttcaaaa
atgtagggccagagtttagccagccagtggtggtgcttgcctttatgcctttaatcccagcactctggaggcagagacaggcagatctctgagtttgagcccagcc
tggtctacacatcaagttctatctaggatagccaggaatacacacagaaaccctgttggggagggggggctctgagatttcataaaattataattgaagcattccc
taatgagccactatggatgtggctaaatccgtctacctttctgatgagatttgggtattattttttctgtctctgctgttggttgggtcttttgacactgtgggctttcttt
aaagcctccttcctgccatgtggtctcttgtttgctactaacttcccatggcttaaatggcatggctttttgccttctaagggcagctgctgagatttgcagcctgattt
ccagggtggggttgggaaatctttcaaacactaaaattgtcctttaattttttttttaaaaaatgggttatataataaacctcataaaatagttatgaggagtgagg
tggactaatattaaatgagtccctcccctataaaagagctattaaggctttttgtcttatacttaactttttttttaaatgtggtatctttagaaccaagggtcttaga
gttttagtatacagaaactgttgcatcgcttaatcagattttctagtttgtgcacaggcctgatatcggtacctccagcttttgttccctttagtgagggttaattgcg
cgcttggcgtaatcatggtcatagctgtttcctgtgtgaaattgttatccgctcacaattccacacaacatacgagccggaagcataaagtgtaaagcctggggtg
cctaatgagtgagctaactcacattaattgcgttgcgctcactgcccgctttccagtcgggaaacctgtcgtgccagctgcattaatgaatcggccaacgcgcggg
gagaggcggtttgcgtattgggcgctcttccgcttcctcgctcactgactcgctgcgctcggtcgttcggctgcggcgagcggtatcagctcactcaaaggcggtaa
tacggttatccacagaatcaggggataacgcaggaaagaacatgtgagcaaaaggccagcaaaaggccaggaaccgtaaaaaggccgcgttgctggcgtttt
tccataggctccgcccccctgacgagcatcacaaaaatcgacgctcaagtcagaggtggcgaaacccgacaggactataaagataccaggcgtttccccctgga
agctccctcgtgcgctctcctgttccgaccctgccgcttaccggatacctgtccgcctttctcccttcgggaagcgtggcgctttctcatagctcacgctgtaggtatct
cagttcggtgtaggtcgttcgctccaagctgggctgtgtgcacgaaccccccgttcagcccgaccgctgcgccttatccggtaactatcgtcttgagtccaacccgg
taagacacgacttatcgccactggcagcagccactggtaacaggattagcagagcgaggtatgtaggcggtgctacagagttcttgaagtggtggcctaactac
ggctacactagaaggacagtatttggtatctgcgctctgctgaagccagttaccttcggaaaaagagttggtagctcttgatccggcaaacaaaccaccgctggt
agcggtggtttttttgtttgcaagcagcagattacgcgcagaaaaaaaggatctcaagaagatcctttgatcttttctacggggtctgacgctcagtggaacgaaa
actcacgttaagggattttggtcatgagattatcaaaaaggatcttcacctagatccttttaaattaaaaatgaagttttaaatcaatctaaagtatatatgagta
aacttggtctgacagttaccaatgcttaatcagtgaggcacctatctcagcgatctgtctatttcgttcatccatagttgcctgactccccgtcgtgtagataactac
gatacgggagggcttaccatctggccccagtgctgcaatgataccgcgagacccacgctcaccggctccagatttatcagcaataaaccagccagccggaaggg
ccgagcgcagaag

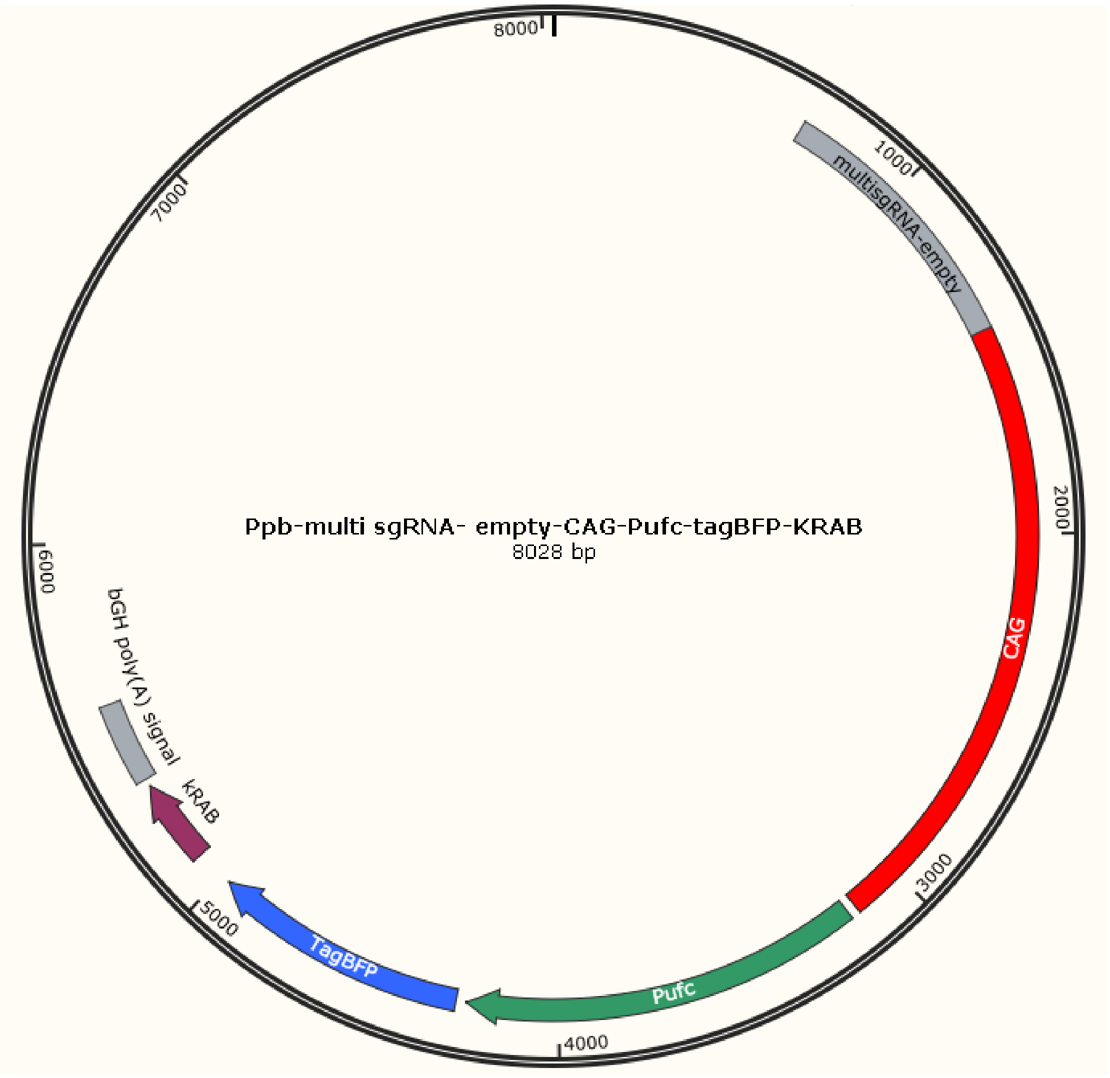

Ppb-multi sgRNA- empty-CAG-PUFc-TagBFP-KRAB
ctaaattgtaagcgttaatattttgttaaaattcgcgttaaatttttgttaaatcagctcattttttaaccaataggccgaaatcggcaaaatcccttataaatcaaa
agaatagaccgagatagggttgagtgttgttccagtttggaacaagagtccactattaaagaacgtggactccaacgtcaaagggcgaaaaaccgtctatcagg
gcgatggcccactacgtgaaccatcaccctaatcaagttttttggggtcgaggtgccgtaaagcactaaatcggaaccctaaagggagcccccgatttagagctt
gacggggaaagccggcgaacgtggcgagaaaggaagggaagaaagcgaaaggagcgggcgctagggcgctggcaagtgtagcggtcacgctgcgcgtaac
caccacacccgccgcgcttaatgcgccgctacagggcgcgtcccattcgccattcaggctgcgcaactgttgggaagggcgatcggtgcgggcctcttcgctatta
cgccagctggcgaaagggggatgtgctgcaaggcgattaagttgggtaacgccagggttttcccagtcacgacgttgtaaaacgacggccagtgagcgcgcgta
atacgactcactatagggcgaattgtctagattaaccctagaaagatagtctgcgtaaaattgacgcatgcattcttgaaatattgctctctctttctaaatagcgc
gaatccgtcgctgtgcatttaggacatctcagtcgccgcttggagctcccgtgaggcgtgcttgtcaatgcggtaagtgtcactgattttgaactataacgaccgcg
tgagtcaaaatgacgcatgattatcttttacgtgacttttaagatttaactcatacgataattatattgttatttcatgttctacttacgtgataacttattatatatat
atttcttgttatagatatcCTCTgGAGACGatggtgagcaagggcgaggagctgttcaccggggtggtgcccatcctggtcgagctggacggcgacgtaaa
cggccacaagttcagcgtgtccggcgagggcgagggcgatgccacctacggcaagctgaccctgaagctgatctgcaccaccggcaagctgcccgtgccctggc
ccaccctcgtgaccaccctgggctacggcctgcagtgcttcgcccgctaccccgaccacatgaagcagcacgacttcttcaagtccgccatgcccgaaggctacgt
ccaggagcgcaccatcttcttcaaggacgacggcaactacaagacccgcgccgaggtgaagttcgagggcgacaccctggtgaaccgcatcgagctgaagggc
atcgacttcaaCGTCTCtAGGTagatcttgagacaaatggcagtattcatccacaaatcaactagaatgctagttattaatagtaatcaattacggggtcatt
agttcatagcccatatatggagttccgcgttacataacttacggtaaatggcccgcctggctgaccgcccaacgacccccgcccattgacgtcaataatgacgtat
gttcccatagtaacgccaatagggactttccattgacgtcaatgggtggactatttacggtaaactgcccacttggcagtacatcaagtgtatcatatgccaagta
cgccccctattgacgtcaatgacggtaaatggcccgcctggcattatgcccagtacatgaccttatgggactttcctacttggcagtacatctacgtattagtcatcg

ctattaccatgggtcgaggtgagccccacgttctgcttcactctccccatctcccccccctccccacccccaattttgtatttatttattttttaattattttgtgcagcg
atgggggcgggggggggggggggcgcgcgccaggcggggcggggcggggcgaggggcggggcggggcgaggcggagaggtgcggcggcagccaatcagagc
ggcgcgctccgaaagtttccttttatggcgaggcggcggcggcggcggccctataaaaagcgaagcgcgcggcgggcgggagtcgctgcgttgccttcgccccgt
gccccgctccgcgccgcctcgcgccgcccgccccggctctgactgaccgcgttactcccacaggtgagcgggcgggacggcccttctcctccgggctgtaattagcg
cttggtttaatgacggctcgtttcttttctgtggctgcgtgaaagccttaaagggctccgggagggccctttgtgcgggggggagcggctcggggggtgcgtgcgtg
tgtgtgtgcgtggggagcgccgcgtgcggcccgcgctgcccggcggctgtgagcgctgcgggcgcggcgcggggctttgtgcgctccgcgtgtgcgcgaggggag
cgcggccgggggcggtgccccgcggtgcgggggggctgcgaggggaacaaaggctgcgtgcggggtgtgtgcgtgggggggtgagcagggggtgtgggcgcg
gcggtcgggctgtaacccccccctgcacccccctccccgagttgctgagcacggcccggcttcgggtgcggggctccgtgcggggcgtggcgcggggctcgccgtg
ccgggcgggggtggcggcaggtgggggtgccgggcggggcggggccgcctcgggccggggagggctcgggggaggggcgcggcggccccggagcgccggc
ggctgtcgaggcgcggcgagccgcagccattgccttttatggtaatcgtgcgagagggcgcagggacttcctttgtcccaaatctggcggagccgaaatctggga
ggcgccgccgcaccccctctagcgggcgcgggcgaagcggtgcggcgccggcaggaaggaaatgggcggggagggccttcgtgcgtcgccgcgccgccgtccc
cttctccatctccagcctcggggctgccgcagggggacggctgccttcgggggggacggggcagggcggggttcggcttctggcgtgtgaccggcggctctagag
cctctgctaaccatgttcatgccttcttctttttcctacagctcctgggcaacgtgctggttattgtgctgtctcatcattttggcaaagaattcggatccaggcgcgcc
GCCACCATGGGGATCCTCCCCCCCAAGAAAAAGAGGAAGGTATCTAGAGGCCGCAGCCGCCTTTTGGAAGATTTTCGAAA
CAACCGGTACCCCAATTTACAACTGCGGGAGATTGCTGGACATATAATGGAATTTTCCCAAGACCAGCATGGGTCCAGATT
CATTCAGCTGAAACTGGAGCGTGCCACACCAGCTGAGCGCCAGCTTGTGTTCAATGAAATCCTCCAGGCTGCCTACCAACT
CATGGTGGATGTGTTTGGTAATTACGTCATTCAGAAGTTCTTTGAATTTGGCAGTCTTGAACAGAAGCTGGCTTTGGCAGA
ACGGATTCGAGGCCACGTCCTGTCATTGGCACTACAGATGTATGGCAGCCGTGTTATCGAGAAAGCTCTTGAGTTTATTCC
TTCAGACCAGCAGAATGAGATGGTTCGGGAACTAGATGGCCATGTCTTGAAGTGTGTGAAAGATCAGAATGGCAATCAC
GTGGTTCAGAAATGCATTGAATGTGTACAGCCCCAGTCTTTGCAATTTATCATCGAcGCGTTTAAGGGACAGGTATTTGCCT
TATCCACACATCCTTATGGCTGCCGAGTGATTCAGAGAATCCTGGAGCACTGTCTCCCTGACCAGACACTCCCTATTTTAGA
GGAGCTTCACCAGCACACAGAGCAGCTGGTACAGGATCAATATGGAAgTTATGTAATCgAACATGTACTGGAGCACGGTC
GTCCTGAGGATAAAAGCAAAATTGTAGCAGAAATCCGAGGCAATGTACTTGTATTGAGTCAGCACAAATTTGCAAaCAAT
GTTGTGcAGAAGTGTGTTACTCACGCCTCACGTACGGAGCGCGCTGTGCTCATCGAcGAGGTGTGCACCATGAACGACGGT
CCCCACAGTGCCTTATACACCATGATGAAGGACCAGTATGCCAACTACGTGGTCCAGAAGATGATTGACGTGGCGGAGCC
AGGCCAGCGGAAGATCGTCATGCATAAGATCCGGCCCCACATCGCAACTCTTCGTAAGTACACCTATGGCAAGCACATTCT
GGCCAAGCTGGAGAAGTACTACATGAAGAACGGTGTTGACTTAGGGTTGGATCCAGGTGGAGGTGGAAGCGGTATGAG
CGAGCTGATTAAGGAGAACATGCACATGAAGCTGTACATGGAGGGCACCGTGGACAACCATCACTTCAAGTGCACATCCG
AGGGCGAAGGCAAGCCCTACGAGGGCACCCAGACCATGAGAATCAAGGTGGTCGAGGGCGGCCCTCTCCCCTTCGCCTTC
GACATCCTGGCTACTAGCTTCCTCTACGGCAGCAAGACCTTCATCAACCACACCCAGGGCATCCCCGACTTCTTCAAGCAGT
CCTTCCCTGAGGGCTTCACATGGGAGAGAGTCACCACATACGAAGACGGGGGCGTGCTGACCGCTACCCAGGACACCAGC
CTCCAGGACGGCTGCCTCATCTACAACGTCAAGATCAGAGGGGTGAACTTCACATCCAACGGCCCTGTGATGCAGAAGAA
AACACTCGGCTGGGAGGCCTTCACCGAGACGCTGTACCCCGCTGACGGCGGCCTGGAAGGCAGAAACGACATGGCCCTG
AAGCTCGTGGGCGGGAGCCATCTGATCGCAAACATCAAGACCACATATAGATCCAAGAAACCCGCTAAGAACCTCAAGAT
GCCTGGCGTCTACTATGTGGACTACAGACTGGAAAGAATCAAGGAGGCCAACAACGAGACCTACGTCGAGCAGCACGAG
GTGGCAGTGGCCAGATACTGCGACCTCCCTAGCAAACTGGGGCACAAGCTTAATGGTGGAGGTCGGACCGGAcccaagaag
aagaggaaggtaggaagtggaagcccaaagaaaaagcggaaagtgggctcaccgaaaaagaagcgtaaggttACCGGTGGCGGTGGCGGAGGG
ATGGATGCTAAGTCACTAACTGCCTGGTCCCGGACACTGGTGACCTTCAAGGATGTATTTGTGGACTTCACCAGGGAGGA
GTGGAAGCTGCTGGACACTGCTCAGCAGATCGTGTACAGAAATGTGATGCTGGAGAACTATAAGAACCTGGTTTCCTTGG
GTTATCAGCTTACTAAGCCAGATGTGATCCTCCGGTTGGAGAAGGGAGAAGAGCCCTAAgCGGCCGCgattaattaactgtgcct
tctagttgccagccatctgttgtttgcccctcccccgtgccttccttgaccctggaaggtgccactcccactgtcctttcctaataaaatgaggaaattgcatcgcatt
gtctgagtaggtgtcattctattctggggggtggggtggggcaggacagcaagggggaggattgggaagacaatagcaggcatgctggggatgcggtgggctct
atggctcgacatactagttaaaagttttgttactttatagaagaaattttgagtttttgttttttttaataaataaataaacataaataaattgtttgttgaatttatt
attagtatgtaagtgtaaatataataaaacttaatatctattcaaattaataaataaacctcgatatacagaccgataaaacacatgcgtcaattttacgcatgat

tatctttaacgtacgtcacaatatgattatctttctagggttaatctagtatacgcgtgcttttgttccctttagtgagggttaattgcgcgcttggcgtaatcatggtc
atagctgtttcctgtgtgaaattgttatccgctcacaattccacacaacatacgagccggaagcataaagtgtaaagcctggggtgcctaatgagtgagctaactc
acattaattgcgttgcgctcactgcccgctttccagtcgggaaacctgtcgtgccagctgcattaatgaatcggccaacgcgcggggagaggcggtttgcgtattg
ggcgctcttccgcttcctcgctcactgactcgctgcgctcggtcgttcggctgcggcgagcggtatcagctcactcaaaggcggtaatacggttatccacagaatca
ggggataacgcaggaaagaacatgtgagcaaaaggccagcaaaaggccaggaaccgtaaaaaggccgcgttgctggcgtttttccataggctccgcccccctg
acgagcatcacaaaaatcgacgctcaagtcagaggtggcgaaacccgacaggactataaagataccaggcgtttccccctggaagctccctcgtgcgctctcct
gttccgaccctgccgcttaccggatacctgtccgcctttctcccttcgggaagcgtggcgctttctcatagctcacgctgtaggtatctcagttcggtgtaggtcgttc
gctccaagctgggctgtgtgcacgaaccccccgttcagcccgaccgctgcgccttatccggtaactatcgtcttgagtccaacccggtaagacacgacttatcgcc
actggcagcagccactggtaacaggattagcagagcgaggtatgtaggcggtgctacagagttcttgaagtggtggcctaactacggctacactagaaggaca
gtatttggtatctgcgctctgctgaagccagttaccttcggaaaaagagttggtagctcttgatccggcaaacaaaccaccgctggtagcggtggtttttttgtttgc
aagcagcagattacgcgcagaaaaaaaggatctcaagaagatcctttgatcttttctacggggtctgacgctcagtggaacgaaaactcacgttaagggatttt
ggtcatgagattatcaaaaaggatcttcacctagatccttttaaattaaaaatgaagttttaaatcaatctaaagtatatatgagtaaacttggtctgacagttac
caatgcttaatcagtgaggcacctatctcagcgatctgtctatttcgttcatccatagttgcctgactccccgtcgtgtagataactacgatacgggagggcttacca
tctggccccagtgctgcaatgataccgcgagacccacgctcaccggctccagatttatcagcaataaaccagccagccggaagggccgagcgcagaagtggtcc
tgcaactttatccgcctccatccagtctattaattgttgccgggaagctagagtaagtagttcgccagttaatagtttgcgcaacgttgttgccattgctacaggcat
cgtggtgtcacgctcgtcgtttggtatggcttcattcagctccggttcccaacgatcaaggcgagttacatgatcccccatgttgtgcaaaaaagcggttagctcctt
cggtcctccgatcgttgtcagaagtaagttggccgcagtgttatcactcatggttatggcagcactgcataattctcttactgtcatgccatccgtaagatgcttttct
gtgactggtgagtactcaaccaagtcattctgagaatagtgtatgcggcgaccgagttgctcttgcccggcgtcaatacgggataataccgcgccacatagcaga
actttaaaagtgctcatcattggaaaacgttcttcggggcgaaaactctcaaggatcttaccgctgttgagatccagttcgatgtaacccactcgtgcacccaact
gatcttcagcatcttttactttcaccagcgtttctgggtgagcaaaaacaggaaggcaaaatgccgcaaaaaagggaataagggcgacacggaaatgttgaat
actcatactcttcctttttcaatattattgaagcatttatcagggttattgtctcatgagcggatacatatttgaatgtatttagaaaaataaacaaataggggttcc
gcgcacatttccccgaaaagtgccac

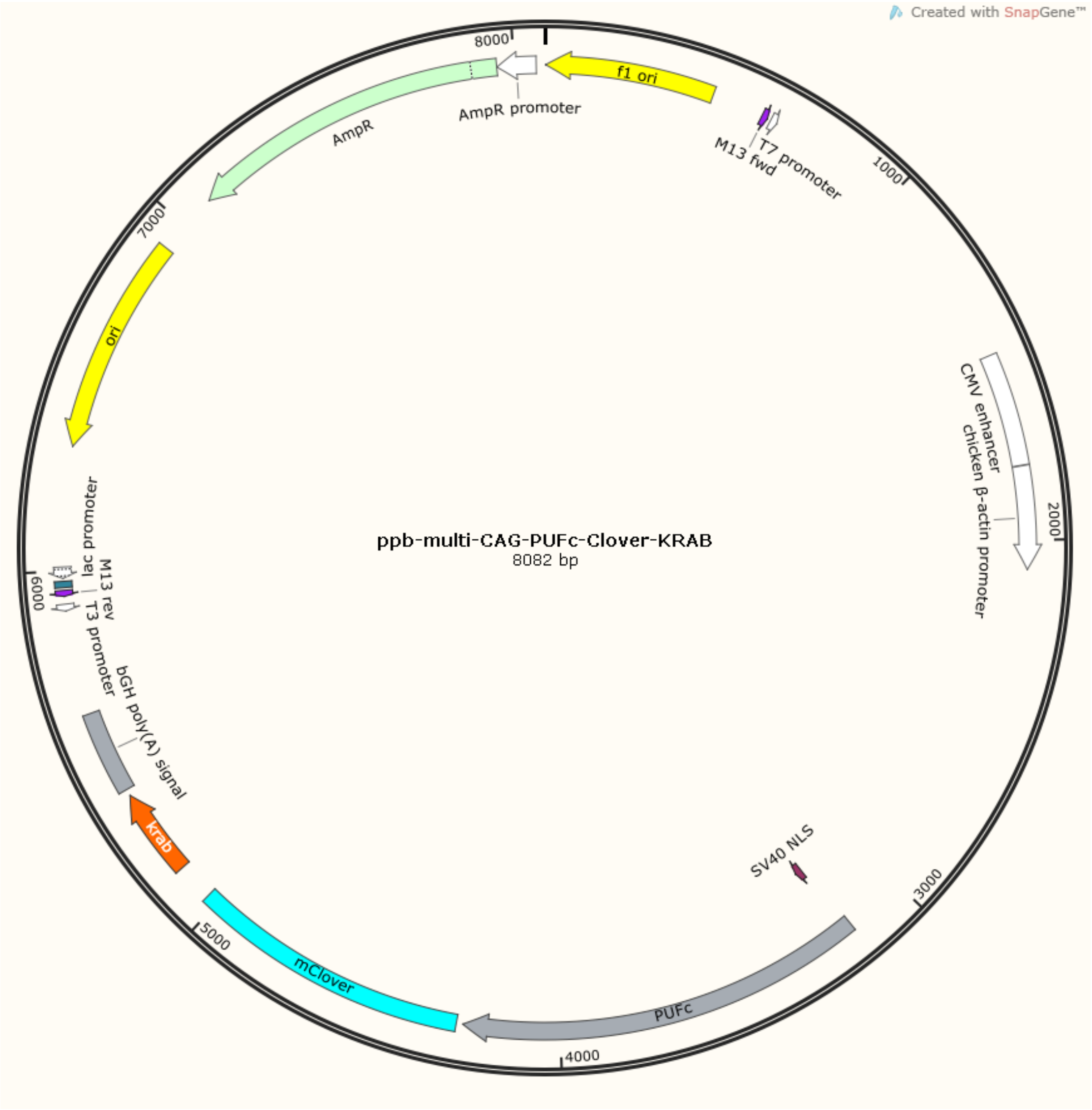

**Ppb-multi sgRNA- empty-CAG-PUFc-Clover-KRAB**
**ctaaattgtaagcgttaatattttgttaaaattcgcgttaaatttttgttaaatcagctcattttttaaccaataggccgaaatcggcaaaatcccttataaatcaaa**
**agaatagaccgagatagggttgagtgttgttccagtttggaacaagagtccactattaaagaacgtggactccaacgtcaaagggcgaaaaaccgtctatcagg**
**gcgatggcccactacgtgaaccatcaccctaatcaagttttttggggtcgaggtgccgtaaagcactaaatcggaaccctaaagggagcccccgatttagagctt**
**gacggggaaagccggcgaacgtggcgagaaaggaagggaagaaagcgaaaggagcgggcgctagggcgctggcaagtgtagcggtcacgctgcgcgtaac**
**caccacacccgccgcgcttaatgcgccgctacagggcgcgtcccattcgccattcaggctgcgcaactgttgggaagggcgatcggtgcgggcctcttcgctatta**
**cgccagctggcgaaagggggatgtgctgcaaggcgattaagttgggtaacgccagggttttcccagtcacgacgttgtaaaacgacggccagtgagcgcgcgta**
**atacgactcactatagggcgaattgtctagattaaccctagaaagatagtctgcgtaaaattgacgcatgcattcttgaaatattgctctctctttctaaatagcgc**
**gaatccgtcgctgtgcatttaggacatctcagtcgccgcttggagctcccgtgaggcgtgcttgtcaatgcggtaagtgtcactgattttgaactataacgaccgcg**
**tgagtcaaaatgacgcatgattatcttttacgtgacttttaagatttaactcatacgataattatattgttatttcatgttctacttacgtgataacttattatatatat**
**attttcttgttatagatatcCTCTgGAGACGatggtgagcaagggcgaggagctgttcaccggggtggtgcccatcctggtcgagctggacggcgacgtaaa**
**cggccacaagttcagcgtgtccggcgagggcgagggcgatgccacctacggcaagctgaccctgaagctgatctgcaccaccggcaagctgcccgtgccctggc**
**ccaccctcgtgaccaccctgggctacggcctgcagtgcttcgcccgctaccccgaccacatgaagcagcacgacttcttcaagtccgccatgcccgaaggctacgt**
**ccaggagcgcaccatcttcttcaaggacgacggcaactacaagacccgcgccgaggtgaagttcgagggcgacaccctggtgaaccgcatcgagctgaagggc**
**atcgacttcaaCGTCTCtAGGTagatcttgagacaaatggcagtattcatccacaatcaactagaatgctagttattaatagtaatcaattacggggtcatt**
**agttcatagcccatatatggagttccgcgttacataacttacggtaaatggcccgcctggctgaccgcccaacgacccccgcccattgacgtcaataatgacgtat**

gttcccatagtaacgccaatagggactttccattgacgtcaatgggtggactatttacggtaaactgcccacttggcagtacatcaagtgtatcatatgccaagta
cgccccctattgacgtcaatgacggtaaatggcccgcctggcattatgcccagtacatgaccttatgggactttcctacttggcagtacatctacgtattagtcatcg
ctattaccatgggtcgaggtgagccccacgttctgcttcactctccccatctcccccccctccccacccccaattttgtatttatttattttttaattattttgtgcagcg
atgggggcgggggggggggggggcgcgcgccaggcggggcggggcggggcgaggggcggggcggggcgaggcggagaggtgcggcggcagccaatcagagc
ggcgcgctccgaaagtttccttttatggcgaggcggcggcggcggcggccctataaaaagcgaagcgcgcggcgggcgggagtcgctgcgttgccttcgccccgt
gccccgctccgcgccgcctcgcgccgcccgccccggctctgactgaccgcgttactcccacaggtgagcgggcgggacggcccttctcctccgggctgtaattagcg
cttggtttaatgacggctcgtttcttttctgtggctgcgtgaaagccttaaagggctccgggagggccctttgtgcggggggggagcggctcggggggtgcgtgcgtg
tgtgtgtgcgtggggagcgccgcgtgcggcccgcgctgcccggcggctgtgagcgctgcgggcgcggcgcggggctttgtgcgctccgcgtgtgcgcgaggggag
cgcggccgggggcggtgccccgcggtgcggggggggctgcgaggggaacaaaggctgcgtgcggggtgtgtgcgtgggggggtgagcagggggtgtgggcgcg
gcggtcggctgtaacccccccctgcacccccctccccgagttgctgagcacggcccggcttcgggtgcggggctccgtgcggggcgtggcgcggggctcgccgtg
ccgggcggggggtggcggcaggtgggggtgccgggcggggcggggccgcctcgggccggggagggctcgggggaggggcgcggcggcccccggagcgccggc
ggctgtcgaggcgcggcgagccgcagccattgccttttatggtaatcgtgcgagagggcgcagggacttcctttgtcccaaatctggcggagccgaaatctggga
ggcgccgccgcaccccctctagcgggcgcgggcgaagcggtgcggcgccggcaggaaggaaatgggcggggagggccttcgtgcgtcgccgcgccgccgtccc
cttctccatctccagcctcggggctgccgcagggggacggctgccttcgggggggacggggcagggcggggttcggcttctggcgtgtgaccggcggctctagag
cctctgctaaccatgttcatgccttcttctttttcctacagctcctgggcaacgtgctggttattgtgctgtctcatcattttggcaaagaattcggatccaggcgcgcc
gccaccATGGGGATCCTCCCCCCCAAGAAAAAGAGGAAGGTATCTAGAGGCCGCAGCCGCCTTTTGGAAGATTTTCGAAAC
AACCGGTACCCCAATTTACAACTGCGGGAGATTGCTGGACATATAATGGAATTTTCCCAAGACCAGCATGGGTCCAGATTC
ATTCAGCTGAAACTGGAGCGTGCCACACCAGCTGAGCGCCAGCTTGTGTTCAATGAAATCCTCCAGGCTGCCTACCAACTC
ATGGTGGATGTGTTTGGTAATTACGTCATTCAGAAGTTCTTTGAATTTGGCAGTCTTGAACAGAAGCTGGCTTTGGCAGAA
CGGATTCGAGGCCACGTCCTGTCATTGGCACTACAGATGTATGGCAGCCGTGTTATCGAGAAAGCTCTTGAGTTTATTCCTT
CAGACCAGCAGAATGAGATGGTTCGGGAACTAGATGGCCATGTCTTGAAGTGTGTGAAAGATCAGAATGGCAATCACGT
GGTTCAGAAATGCATTGAATGTGTACAGCCCCAGTCTTTGCAATTTATCATCGAcGCGTTTAAGGGACAGGTATTTGCCTTA
TCCACACATCCTTATGGCTGCCGAGTGATTCAGAGAATCCTGGAGCACTGTCTCCCTGACCAGACACTCCCTATTTTAGAGG
AGCTTCACCAGCACACAGAGCAGCTGGTACAGGATCAATATGGAAgTTATGTAATCgAACATGTACTGGAGCACGGTCGT
CCTGAGGATAAAAGCAAAATTGTAGCAGAAATCCGAGGCAATGTACTTGTATTGAGTCAGCACAAATTTGCAAaCAATGT
TGTGcAGAAGTGTGTTACTCACGCCTCACGTACGGAGCGCGCTGTGCTCATCGAcGAGGTGTGCACCATGAACGACGGTCC
CCACAGTGCCTTATACACCATGATGAAGGACCAGTATGCCAACTACGTGGTCCAGAAGATGATTGACGTGGCGGAGCCAG
GCCAGCGGAAGATCGTCATGCATAAGATCCGGCCCCACATCGCAACTCTTCGTAAGTACACCTATGGCAAGCACATTCTGG
CCAAGCTGGAGAAGTACTACATGAAGAACGGTGTTGACTTAGGGTTGGATCCAGGTGGAGGTGGAAGCGGTATGGTGA
GCAAGGGCGAGGAGCTGTTCACCGGGGTGGTGCCCATCCTGGTCGAGCTGGACGGCGACGTAAACGGCCACAAGTTCAG
CGTCCGCGGCGAGGGCGAGGGCGATGCCACCAACGGCAAGCTGACCCTGAAGTTCATCTGCACCACCGGCAAGCTGCCCG
TGCCCTGGCCCACCCTCGTGACCACCTTCGGCTACGGCGTGGCCTGCTTCAGCCGCTACCCCGACCACATGAAGCAGCACG
ACTTCTTCAAGTCCGCCATGCCCGAAGGCTACGTCCAGGAGCGCACCATCTCTTTCAAGGACGACGGTACCTACAAGACCC
GCGCCGAGGTGAAGTTCGAGGGCGACACCCTGGTGAACCGCATCGAGCTGAAGGGCATCGACTTCAAGGAGGACGGCAA
CATCCTGGGGCACAAGCTGGAGTACAACTTCAACAGCCACAACGTCTATATCACGGCCGACAAGCAGAAGAACGGCATCA
AGGCTAACTTCAAGATCCGCCACAACGTTGAGGACGGCAGCGTGCAGCTCGCCGACCACTACCAGCAGAACACCCCCATC
GGCGACGGCCCCGTGCTGCTGCCCGACAACCACTACCTGAGCCATCAGTCCGCCCTGAGCAAAGACCCCAACGAGAAGCG
CGATCACATGGTCCTGCTGGAGTTCGTGACCGCCGCCGGGATTACACATGGCATGGACGAGCTGTACAAGTCTAGAGGGC
CCTATTCTATAGTGTCACCTAAATGCGGTGGAGGTCGGACCGGACCCAAGAAGAAGAGGAAGGTAGGAAGTGGAAGCCC
AAAGAAAAAGCGGAAAGTGGGCTCACCGAAAAAGAAGCGTAAGGTTACCGGTGGCGGTGGCGGAGGGATGGATGCTA
AGTCACTAACTGCCTGGTCCCGGACACTGGTGACCTTCAAGGATGTATTTGTGGACTTCACCAGGGAGGAGTGGAAGCTG
CTGGACACTGCTCAGCAGATCGTGTACAGAAATGTGATGCTGGAGAACTATAAGAACCTGGTTTCCTTGGGTTATCAGCTT
ACTAAGCCAGATGTGATCCTCCGGTTGGAGAAGGGAGAAGAGCCCTAAgcggccgcgattaattaactgtgccttctagttgccagccat
ctgttgtttgcccctcccccgtgccttccttgaccctggaaggtgccactcccactgtcctttcctaataaaatgaggaaattgcatcgcattgtctgagtaggtgtca

ttctattctggggggtggggtggggcaggacagcaagggggaggattgggaagacaatagcaggcatgctggggatgcggtgggctctatggctcgacatact

agttaaaagttttgttactttatagaagaaattttgagtttttgttttttttaataaataaataaacataaataaattgtttgttgaatttattattagtatgtaagtg

taaatataataaaacttaatatctattcaaattaataaataaacctcgatatacagaccgataaaacacatgcgtcaattttacgcatgattatctttaacgtacg

tcacaatatgattatctttctagggttaatctagtatacgcgtgcttttgttccctttagtgagggttaattgcgcgcttggcgtaatcatggtcatagctgtttcctgt

gtgaaattgttatccgctcacaattccacacaacatacgagccggaagcataaagtgtaaagcctggggtgcctaatgagtgagctaactcacattaattgcgtt

gcgctcactgcccgctttccagtcgggaaacctgtcgtgccagctgcattaatgaatcggccaacgcgcggggagaggcggtttgcgtattgggcgctcttccgctt

cctcgctcactgactcgctgcgctcggtcgttcggctgcggcgagcggtatcagctcactcaaaggcggtaatacggttatccacagaatcaggggataacgcag

gaaagaacatgtgagcaaaaggccagcaaaaggccaggaaccgtaaaaaggccgcgttgctggcgtttttccataggctccgcccccctgacgagcatcacaa

aaatcgacgctcaagtcagaggtggcgaaacccgacaggactataaagataccaggcgtttccccctggaagctccctcgtgcgctctcctgttccgaccctgcc

gcttaccggatacctgtccgcctttctcccttcgggaagcgtggcgctttctcatagctcacgctgtaggtatctcagttcggtgtaggtcgttcgctccaagctgggc

tgtgtgcacgaaccccccgttcagcccgaccgctgcgccttatccggtaactatcgtcttgagtccaacccggtaagacacgacttatcgccactggcagcagcca

ctggtaacaggattagcagagcgaggtatgtaggcggtgctacagagttcttgaagtggtggcctaactacggctacactagaaggacagtatttggtatctgcg

ctctgctgaagccagttaccttcggaaaaagagttggtagctcttgatccggcaaacaaaccaccgctggtagcggtggtttttttgtttgcaagcagcagattac

gcgcagaaaaaaaggatctcaagaagatcctttgatcttttctacggggtctgacgctcagtggaacgaaaactcacgttaagggattttggtcatgagattatc

aaaaaggatcttcacctagatccttttaaattaaaaatgaagttttaaatcaatctaaagtatatatgagtaaacttggtctgacagttaccaatgcttaatcagt

gaggcacctatctcagcgatctgtctatttcgttcatccatagttgcctgactccccgtcgtgtagataactacgatacgggagggcttaccatctggccccagtgct

gcaatgataccgcgagacccacgctcaccggctccagatttatcagcaataaaccagccagccggaagggccgagcgcagaagtggtcctgcaactttatccgc

ctccatccagtctattaattgttgccgggaagctagagtaagtagttcgccagttaatagtttgcgcaacgttgttgccattgctacaggcatcgtggtgtcacgctc

gtcgtttggtatggcttcattcagctccggttcccaacgatcaaggcgagttacatgatcccccatgttgtgcaaaaaagcggttagctccttcggtcctccgatcgt

tgtcagaagtaagttggccgcagtgttatcactcatggttatggcagcactgcataattctcttactgtcatgccatccgtaagatgcttttctgtgactggtgagta

ctcaaccaagtcattctgagaatagtgtatgcggcgaccgagttgctcttgcccggcgtcaatacgggataataccgcgccacatagcagaactttaaaagtgct

catcattggaaaacgttcttcggggcgaaaactctcaaggatcttaccgctgttgagatccagttcgatgtaacccactcgtgcacccaactgatcttcagcatctt

ttactttcaccagcgtttctgggtgagcaaaaacaggaaggcaaaatgccgcaaaaaagggaataagggcgacacggaaatgttgaatactcatactcttcctt

tttcaatattattgaagcatttatcagggttattgtctcatgagcggatacatatttgaatgtatttagaaaaataaacaaataggggttccgcgcacatttccccg

aaaagtgccac

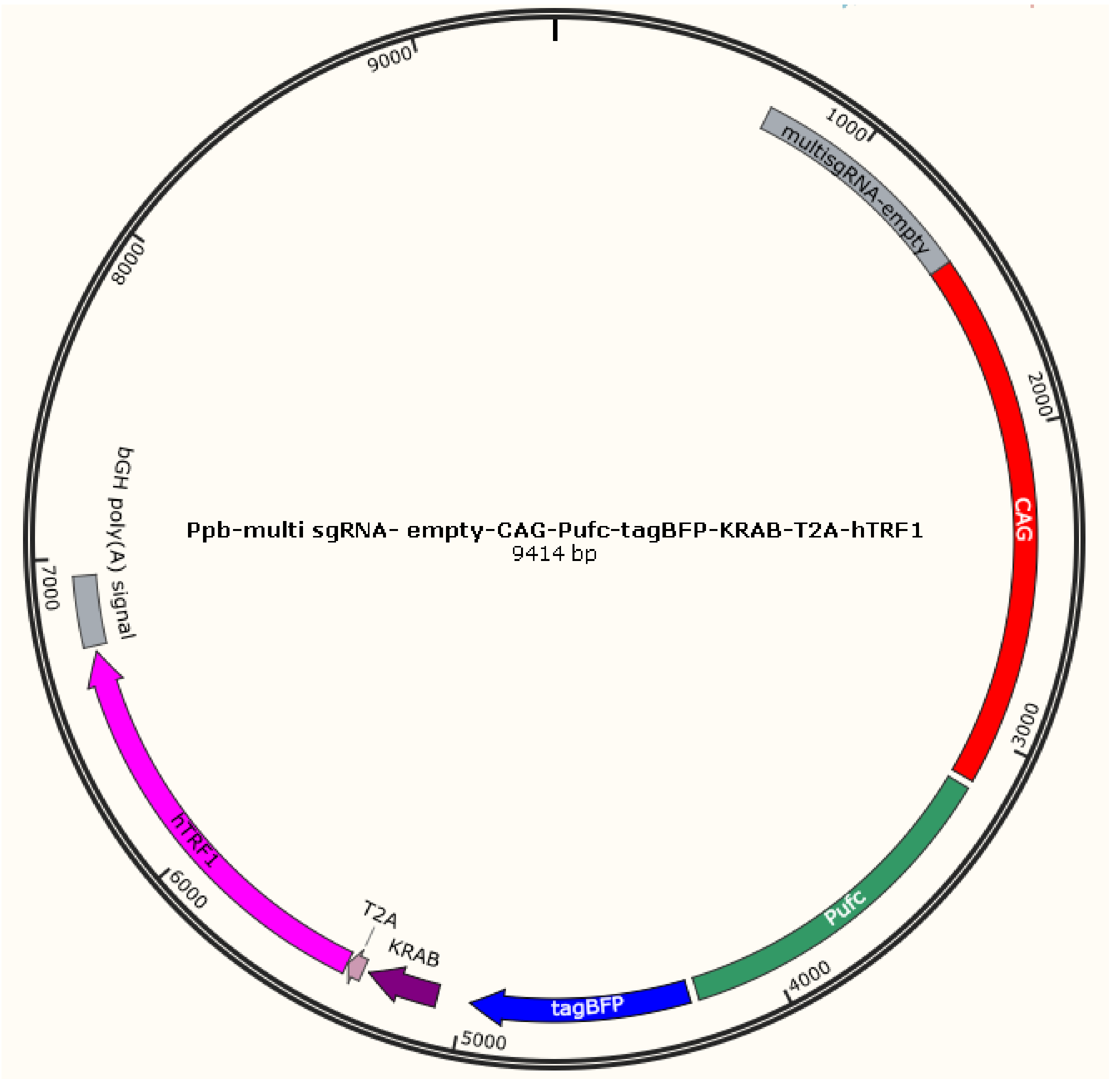

**Ppb-multi sgRNA- empty-CAG-Pufc-TagBFP-KRAB-T2A-hTRF1**
ctaaattgtaagcgttaatattttgttaaaattcgcgttaaatttttgttaaatcagctcattttttaaccaataggccgaaatcggcaaaatcccttataaatcaaa
agaatagaccgagatagggttgagtgttgttccagtttggaacaagagtccactattaaagaacgtggactccaacgtcaaagggcgaaaaaccgtctatcagg
gcgatggcccactacgtgaaccatcaccctaatcaagttttttggggtcgaggtgccgtaaagcactaaatcggaaccctaaagggagcccccgatttagagctt
gacggggaaagccggcgaacgtggcgagaaaggaagggaagaaagcgaaaggagcgggcgctagggcgctggcaagtgtagcggtcacgctgcgcgtaac
caccacacccgccgcgcttaatgcgccgctacagggcgcgtcccattcgccattcaggctgcgcaactgttgggaagggcgatcggtgcgggcctcttcgctatta
cgccagctggcgaaagggggatgtgctgcaaggcgattaagttgggtaacgccagggttttcccagtcacgacgttgtaaaacgacggccagtgagcgcgcgta
atacgactcactatagggcgaattgtctagattaaccctagaaagatagtctgcgtaaaattgacgcatgcattcttgaaatattgctctctctttctaaatagcgc
gaatccgtcgctgtgcatttaggacatctcagtcgccgcttggagctcccgtgaggcgtgcttgtcaatgcggtaagtgtcactgattttgaactataacgaccgcg
tgagtcaaaatgacgcatgattatcttttacgtgacttttaagatttaactcatacgataattatattgttatttcatgttctacttacgtgataacttattatatatat
atttcttgttatagatatcCTCTgGAGACGatggtgagcaagggcgaggagctgttcaccggggtggtgcccatcctggtcgagctggacggcgacgtaaa
cggccacaagttcagcgtgtccggcgagggcgagggcgatgccacctacggcaagctgaccctgaagctgatctgcaccaccggcaagctgcccgtgccctggc
ccaccctcgtgaccaccctgggctacggcctgcagtgcttcgcccgctaccccgaccacatgaagcagcacgacttcttcaagtccgccatgcccgaaggctacgt
ccaggagcgcaccatcttcttcaaggacgacggcaactacaagacccgcgccgaggtgaagttcgagggcgacaccctggtgaaccgcatcgagctgaagggc
atcgacttcaaCGTCTCtAGGTagatcttgagacaaatggcagtattcatccacaatcaactagaatgctagttattaatagtaatcaattacggggtcatt
agttcatagcccatatatggagttccgcgttacataacttacggtaaatggcccgcctggctgaccgcccaacgacccccgcccattgacgtcaataatgacgtat
gttcccatagtaacgccaatagggactttccattgacgtcaatgggtggactatttacggtaaactgcccacttggcagtacatcaagtgtatcatatgccaagta

cgccccctattgacgtcaatgacggtaaatggcccgcctggcattatgcccagtacatgaccttatgggactttcctacttggcagtacatctacgtattagtcatcg
ctattaccatgggtcgaggtgagccccacgttctgcttcactctccccatctcccccccctccccacccccaattttgtatttatttattttttaattattttgtgcagcg
atgggggcgggggggggggggggcgcgcgccaggcggggcggggcggggcgaggggcggggcggggcgaggcggagaggtgcggcggcagccaatcagagc
ggcgcgctccgaaagtttccttttatggcgaggcggcggcggcggcggccctataaaaagcgaagcgcgcggcgggcgggagtcgctgcgttgccttcgccccgt
gccccgctccgcgccgcctcgcgccgcccgccccggctctgactgaccgcgttactcccacaggtgagcgggcgggacggcccttctcctccgggctgtaattagcg
cttggtttaatgacggctcgtttcttttctgtggctgcgtgaaagccttaaagggctccgggagggccctttgtgcggggggagcggctcggggggtgcgtgcgtg
tgtgtgtgcgtggggagcgccgcgtgcggcccgcgctgcccggcggctgtgagcgctgcgggcgcggcgcggggctttgtgcgctccgcgtgtgcgcgaggggag
cgcggccgggggcggtgccccgcggtgcgggggggctgcgaggggaacaaaggctgcgtgcggggtgtgtgcgtgggggggtgagcagggggtgtgggcgcg
gcggtcgggctgtaacccccccctgcacccccctccccgagttgctgagcacggcccggcttcgggtgcggggctccgtgcggggcgtggcgcggggctcgccgtg
ccgggcgggggggtggcggcaggtgggggtgccgggcggggcggggccgcctcgggccggggagggctcgggggaggggcgcggcggccccggagcgccggc
ggctgtcgaggcgcggcgagccgcagccattgccttttatggtaatcgtgcgagagggcgcagggacttcctttgtcccaaatctggcggagccgaaatctggga
ggcgccgccgcacccccctctagcgggcgcgggcgaagcggtgcggcgccggcaggaaggaaatgggcggggagggccttcgtgcgtcgccgcgccgccgtccc
cttctccatctccagcctcggggctgccgcagggggacggctgccttcgggggggacggggcagggcggggttcggcttctggcgtgtgaccggcggctctagag
cctctgctaaccatgttcatgccttcttctttttcctacagctcctgggcaacgtgctggttattgtgctgtctcatcattttggcaaagaattcggatccaggcgcgcc
GCCACCATGGGGATCCTCCCCCCCAAGAAAAAGAGGAAGGTATCTAGAGGCCGCAGCCGCCTTTTGGAAGATTTCGAAA
CAACCGGTACCCCAATTTACAACTGCGGGAGATTGCTGGACATATAATGGAATTTTCCCAAGACCAGCATGGGTCCAGATT
CATTCAGCTGAAACTGGAGCGTGCCACACCAGCTGAGCGCCAGCTTGTGTTCAATGAAATCCTCCAGGCTGCCTACCAACT
CATGGTGGATGTGTTTGGTAATTACGTCATTCAGAAGTTCTTTGAATTTGGCAGTCTTGAACAGAAGCTGGCTTTGGCAGA
ACGGATTCGAGGCCACGTCCTGTCATTGGCACTACAGATGTATGGCAGCCGTGTTATCGAGAAAGCTCTTGAGTTTATTCC
TTCAGACCAGCAGAATGAGATGGTTCGGGAACTAGATGGCCATGTCTTGAAGTGTGTGAAAGATCAGAATGGCAATCAC
GTGGTTCAGAAATGCATTGAATGTGTACAGCCCCAGTCTTTGCAATTTATCATCGAcGCGTTTAAGGGACAGGTATTTGCCT
TATCCACACATCCTTATGGCTGCCGAGTGATTCAGAGAATCCTGGAGCACTGTCTCCCTGACCAGACACTCCCTATTTTAGA
GGAGCTTCACCAGCACACAGAGCAGCTGGTACAGGATCAATATGGAAgTTATGTAATCgAACATGTACTGGAGCACGGTC
GTCCTGAGGATAAAAGCAAAATTGTAGCAGAAATCCGAGGCAATGTACTTGTATTGAGTCAGCACAAATTTGCAAaCAAT
GTTGTGcAGAAGTGTGTTACTCACGCCTCACGTACGGAGCGCGCTGTGCTCATCGAcGAGGTGTGCACCATGAACGACGGT
CCCCACAGTGCCTTATACACCATGATGAAGGACCAGTATGCCAACTACGTGGTCCAGAAGATGATTGACGTGGCGGAGCC
AGGCCAGCGGAAGATCGTCATGCATAAGATCCGGCCCCACATCGCAACTCTTCGTAAGTACACCTATGGCAAGCACATTCT
GGCCAAGCTGGAGAAGTACTACATGAAGAACGGTGTTGACTTAGGGTTGGATCCAGGTGGAGGTGGAAGCGGTATGAG
CGAGCTGATTAAGGAGAACATGCACATGAAGCTGTACATGGAGGGCACCGTGGACAACCATCACTTCAAGTGCACATCCG
AGGGCGAAGGCAAGCCCTACGAGGGCACCCAGACCATGAGAATCAAGGTGGTCGAGGGCGGCCCTCTCCCCTTCGCCTTC
GACATCCTGGCTACTAGCTTCCTCTACGGCAGCAAGACCTTCATCAACCACACCCAGGGCATCCCCGACTTCTTCAAGCAGT
CCTTCCCTGAGGGCTTCACATGGGAGAGAGTCACCACATACGAAGACGGGGGCGTGCTGACCGCTACCCAGGACACCAGC
CTCCAGGACGGCTGCCTCATCTACAACGTCAAGATCAGAGGGGTGAACTTCACATCCAACGGCCCTGTGATGCAGAAGAA
AACACTCGGCTGGGAGGCCTTCACCGAGACGCTGTACCCCGCTGACGGCGGCCTGGAAGGCAGAAACGACATGGCCCTG
AAGCTCGTGGGCGGGAGCCATCTGATCGCAAACATCAAGACCACATATAGATCCAAGAAACCCGCTAAGAACCTCAAGAT
GCCTGGCGTCTACTATGTGGACTACAGACTGGAAAGAATCAAGGAGGCCAACAACGAGACCTACGTCGAGCAGCACGAG
GTGGCAGTGGCCAGATACTGCGACCTCCCTAGCAAACTGGGGCACAAGCTTAATGGTGGAGGTCGGACCGGAcccaagaag
aagaggaaggtaggaagtggaagcccaaagaaaaagcggaaagtgggctcaccgaaaaagaagcgtaaggttACCGGTGGCGGTGGCGGAGGG
ATGGATGCTAAGTCACTAACTGCCTGGTCCCGGACACTGGTGACCTTCAAGGATGTATTTGTGGACTTCACCAGGGAGGA
GTGGAAGCTGCTGGACACTGCTCAGCAGATCGTGTACAGAAATGTGATGCTGGAGAACTATAAGAACCTGGTTTCCTTGG
GTTATCAGCTTACTAAGCCAGATGTGATCCTCCGGTTGGAGAAGGGAGAAGAGCCCggcggcgggtccggaggagagggcagagg
aagtcttctaacatgcggtgacgtggaggagaatcctggcccagcggaggatgtttcctcagcggccccgagcccgcggggctgtgcggatggtagggatgccg
accctactgaggagcagatggcagaaacagagagaaacgacgaggagcagttcgaatgccaggaactgctcgagtgccaggtgcaggtgggggcccccgag
gaggaggaggaggaggaggaggacgcgggcctggtggccgaggccgaggccgtggctgccggctggatgctcgatttcctctgcctctctctttgccgagctttc

cgcgacggccgctccgaggacttccgcaggacccgcaacagcgcagaggctattattcatggactatccagtctaacagcttgccagttgagaacgatatacata
tgtcagtttttgacaagaattgcagcaggaaaaacccttgatgcacagtttgaaaatgatgaacgaattacacccttggaatcagccctgatgatttggggttca
attgaaaaggaacatgacaaacttcatgaagaaatacagaatttaattaaaattcaggctatagctgtttgtatggaaaatggcaactttaaagaagcagaag
aagtctttgaaagaatatttggtgatccaaattctcatatgcctttcaaaagcaaattgcttatgataatctctcagaaagatacatttcattcctttttcaacactt
cagctacaaccacatgatggagaaaattaagagttatgtgaattatgtgctaagtgaaaaatcatcaacctttctaatgaaggcagcggcaaaagtagtagaa
agcaaaaggacaagaacaataacttctcaagataaacctagtggtaatgatgttgaaatggaaactgaagctaatttggatacaagaaaaagtgttagtgaca
aacagtctgcggtaactgaatcctcagagggtacagtatccttattgaggtctcacaagaatcttttcttatctaagttgcaacatggaacccagcaacaagacct
taataagaaagaaagaagagtaggaactcctcaaagtacaaaaaagaaaaagaaagcagaagagccactgaaagcagaatacctgtttcaaagagtcag
ccggtaactcctgaaaaacatcgagctagaaaaagacaggcatggctttgggaagaagacaagaatttgagatctggcgtgaggaaatatggagagggaaac
tggtctaaaatactgttgcattataaattcaacaaccggacaagtgtcatgttaaaagacagatggaggaccatgaagaaactaaaactgatttcctcagacag
cgaagactgagCGGCCGCgattaattaactgtgccttctagttgccagccatctgttgtttgcccctcccccgtgccttccttgaccctggaaggtgccactccca
ctgtcctttcctaataaaatgaggaaattgcatcgcattgtctgagtaggtgtcattctattctggggggtggggtggggcaggacagcaagggggaggattggg
aagacaatagcaggcatgctggggatgcggtgggctctatggctcgacatactagttaaaagttttgttactttatagaagaaattttgagtttttgtttttttttaa
taaataaataaacataaataaattgtttgttgaatttattattagtatgtaagtgtaaatataataaaacttaatatctattcaaattaataaataaacctcgatat
acagaccgataaaacacatgcgtcaattttacgcatgattatctttaacgtacgtcacaatatgattatctttctagggttaatctagtatacgcgtgcttttgttcc
ctttagtgagggttaattgcgcgcttggcgtaatcatggtcatagctgtttcctgtgtgaaattgttatccgctcacaattccacacaacatacgagccggaagcat
aaagtgtaaagcctggggtgcctaatgagtgagctaactcacattaattgcgttgcgctcactgcccgctttccagtcgggaaacctgtcgtgccagctgcattaa
tgaatcggccaacgcgcggggagaggcggtttgcgtattgggcgctcttccgcttcctcgctcactgactcgctgcgctcggtcgttcggctgcggcgagcggtatc
agctcactcaaaggcggtaatacggttatccacagaatcaggggataacgcaggaaagaacatgtgagcaaaaggccagcaaaaggccaggaaccgtaaaa
aggccgcgttgctggcgtttttccataggctccgcccccctgacgagcatcacaaaaatcgacgctcaagtcagaggtggcgaaacccgacaggactataaaga
taccaggcgtttccccctggaagctccctcgtgcgctctcctgttccgaccctgccgcttaccggatacctgtccgcctttctcccttcgggaagcgtggcgctttctca
tagctcacgctgtaggtatctcagttcggtgtaggtcgttcgctccaagctgggctgtgtgcacgaaccccccgttcagcccgaccgctgcgccttatccggtaact
atcgtcttgagtccaacccggtaagacacgacttatcgccactggcagcagccactggtaacaggattagcagagcgaggtatgtaggcggtgctacagagttct
tgaagtggtggcctaactacggctacactagaaggacagtatttggtatctgcgctctgctgaagccagttaccttcggaaaaagagttggtagctcttgatccgg
caaacaaaccaccgctggtagcggtggtttttttgtttgcaagcagcagattacgcgcagaaaaaaaggatctcaagaagatcctttgatcttttctacggggtct
gacgctcagtggaacgaaaactcacgttaagggattttggtcatgagattatcaaaaaggatcttcacctagatccttttaaattaaaaatgaagttttaaatca
atctaaagtatatatgagtaaacttggtctgacagttaccaatgcttaatcagtgaggcacctatctcagcgatctgtctatttcgttcatccatagttgcctgactc
cccgtcgtgtagataactacgatacgggagggcttaccatctggccccagtgctgcaatgataccgcgagacccacgctcaccggctccagatttatcagcaata
aaccagccagccggaagggccgagcgcagaagtggtcctgcaactttatccgcctccatccagtctattaattgttgccgggaagctagagtaagtagttcgcca
gttaatagtttgcgcaacgttgttgccattgctacaggcatcgtggtgtcacgctcgtcgtttggtatggcttcattcagctccggttcccaacgatcaaggcgagtt
acatgatcccccatgttgtgcaaaaaagcggttagctccttcggtcctccgatcgttgtcagaagtaagttggccgcagtgttatcactcatggttatggcagcact
gcataattctcttactgtcatgccatccgtaagatgcttttctgtgactggtgagtactcaaccaagtcattctgagaatagtgtatgcggcgaccgagttgctcttg
cccggcgtcaatacgggataataccgcgccacatagcagaactttaaaagtgctcatcattggaaaacgttcttcggggcgaaaactctcaaggatcttaccgct
gttgagatccagttcgatgtaacccactcgtgcacccaactgatcttcagcatcttttactttcaccagcgtttctgggtgagcaaaaacaggaaggcaaaatgcc
gcaaaaaagggaataagggcgacacggaaatgttgaatactcatactcttcctttttcaatattattgaagcatttatcagggttattgtctcatgagcggataca
tatttgaatgtatttagaaaaataaacaaataggggttccgcgcacatttccccgaaaagtgccac

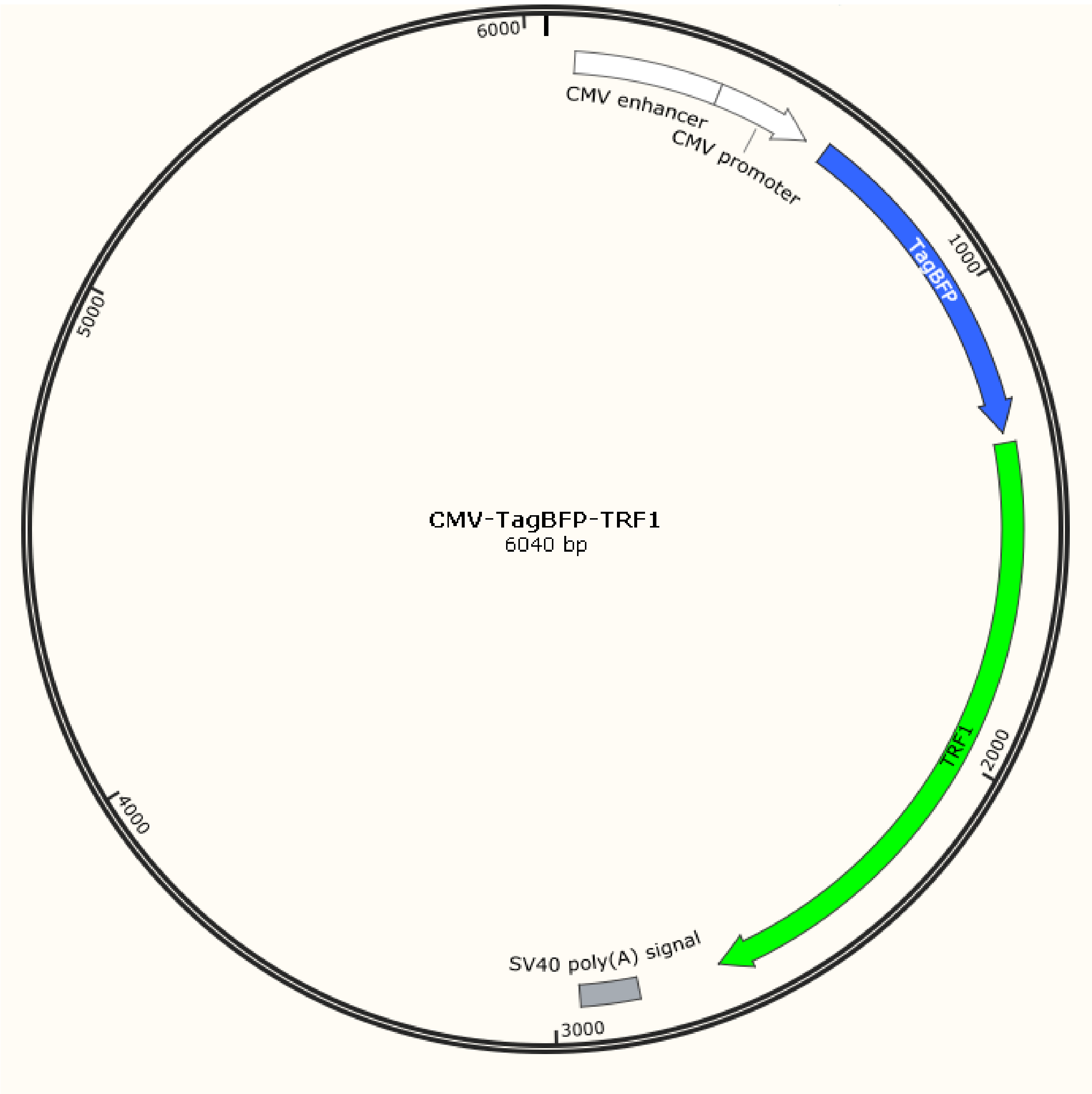

**CMV-TagBFP-TRF1**
**TAGTTATTAATAGTAATCAATTACGGGGTCATTAGTTCATAGCCCATATATGGAGTTCCGCGTTACATAACTTACGGTAAAT**
**GGCCCGCCTGGCTGACCGCCCAACGACCCCCGCCCATTGACGTCAATAATGACGTATGTTCCCATAGTAACGCCAATAGGG**
**ACTTTCCATTGACGTCAATGGGTGGAGTATTTACGGTAAACTGCCCACTTGGCAGTACATCAAGTGTATCATATGCCAAGT**
**ACGCCCCCTATTGACGTCAATGACGGTAAATGGCCCGCCTGGCATTATGCCCAGTACATGACCTTATGGGACTTTCCTACTT**
**GGCAGTACATCTACGTATTAGTCATCGCTATTACCATGGTGATGCGGTTTTGGCAGTACATCAATGGGCGTGGATAGCGGT**
**TTGACTCACGGGGATTTCCAAGTCTCCACCCCATTGACGTCAATGGGAGTTTGTTTTGGCACCAAAATCAACGGGACTTTCC**
**AAAATGTCGTAACAACTCCGCCCCATTGACGCAAATGGGCGGTAGGCGTGTACGGTGGGAGGTCTATATAAGCAGAGCT**
**GGTTTAGTGAACCGTCAGATCCGCTAGCGCTACCGGTcgccaccATGAGCGAGCTGATTAAGGAGAACATGCACATGAAGC**
**TGTACATGGAGGGCACCGTGGACAACCATCACTTCAAGTGCACATCCGAGGGCGAAGGCAAGCCCTACGAGGGCACCCA**
**GACCATGAGAATCAAGGTGGTCGAGGGCGGCCCTCTCCCCTTCGCCTTCGACATCCTGGCTACTAGCTTCCTCTACGGCAGC**
**AAGACCTTCATCAACCACACCCAGGGCATCCCCGACTTCTTCAAGCAGTCCTTCCCTGAGGGCTTCACATGGGAGAGAGTC**
**ACCACATACGAAGACGGGGGCGTGCTGACCGCTACCCAGGACACCAGCCTCCAGGACGGCTGCCTCATCTACAACGTCAA**
**GATCAGAGGGGTGAACTTCACATCCAACGGCCCTGTGATGCAGAAGAAAACACTCGGCTGGGAGGCCTTCACCGAGACG**
**CTGTACCCCGCTGACGGCGGCCTGGAAGGCAGAAACGACATGGCCCTGAAGCTCGTGGGCGGGAGCCATCTGATCGCAA**
**ACATCAAGACCACATATAGATCCAAGAAACCCGCTAAGAACCTCAAGATGCCTGGCGTCTACTATGTGGACTACAGACTG**
**GAAAGAATCAAGGAGGCCAACAACGAGACCTACGTCGAGCAGCACGAGGTGGCAGTGGCCAGATACTGCGACCTCCCTA**

**GCAAACTGGGGCACAAGCTTAATtccGGACTCAGATCTCGAGCTcaaattgcggaggatgtttcctcagcggccccgagcccgcggggctgtg
cggatggtagggatgccgaccctactgaggagcagatggcagaaacagagagaaacgacgaggagcagttcgaatgccaggaactgctcgagtgccaggtgc
aggtgggggcccccgaggaggaggaggaggaggaggaggacgcgggcctggtggccgaggccgaggccgtggctgccggctggatgctcgatttcctctgcct
ctctctttgccgagctttccgcgacggccgctccgaggacttccgcaggacccgcaacagcgcagaggctattattcatggactatccagtctaacagcttgccagt
tgagaacgatatacatatgtcagtttttgacaagaattgcagcaggaaaaacccttgatgcacagtttgaaaatgatgaacgaattacacccttggaatcagccc
tgatgatttggggttcaattgaaaaggaacatgacaaacttcatgaagaaatacagaatttaattaaaattcaggctatagctgtttgtatggaaaatggcaact
ttaaagaagcagaagaagtctttgaaagaatatttggtgatccaaattctcatatgcctttcaaaagcaaattgcttatgataatctctcagaaagatacatttca
ttcctttttcaacacttcagctacaaccacatgatggagaaaattaagagttatgtgaattatgtgctaagtgaaaaatcatcaacctttctaatgaaggcagcg
gcaaaagtagtagaaagcaaaaggacaagaacaataacttctcaagataaacctagtggtaatgatgttgaaatggaaactgaagctaatttggatacaaga
aaaagtgttagtgacaaacagtctgcggtaactgaatcctcagagggtacagtatccttattgaggtctcacaagaatcttttcttatctaagttgcaacatggaa
cccagcaacaagaccttaataagaaagaaagaagagtaggaactcctcaaagtacaaaaaagaaaaaagaaagcagaagagccactgaaagcagaatac
ctgtttcaaagagtcagccggtaactcctgaaaaacatcgagctagaaaaagacaggcatggctttgggaagaagacaagaatttgagatctggcgtgaggaa
atatggagagggaaactggtctaaaatactgttgcattataaattcaacaaccggacaagtgtcatgttaaaagacagatggaggaccatgaagaaactaaaa
ctgatttcctcagacagcgaagactgaAGCTCAAGCTTCGAATTCTGCAGTCGAGGGTACCGCGGGCCCGGGATCCACCGGATCTA
GATAACTGATCATAATCAGCCATACCACATTTGTAGAGGTTTTACTTGCTTTAAAAAACCTCCCACACCTCCCCCTGAACCTG
AAACATAAAATGAATGCAATTGTTGTTGTTAACTTGTTTATTGCAGCTTATAATGGTTACAAATAAAGCAATAGCATCACA
AATTTCACAAATAAAGCATTTTTTTCACTGCATTCTAGTTGTGGTTTGTCCAAACTCATCAATGTATCTTAACGCGTAAATTG
TAAGCGTTAATATTTTGTTAAAATTCGCGTTAAATTTTTGTTAAATCAGCTCATTTTTTAACCAATAGGCCGAAATCGGCAA
AATCCCTTATAAATCAAAAGAATAGACCGAGATAGGGTTGAGTGTTGTTCCAGTTTGGAACAAGAGTCCACTATTAAAGA
ACGTGGACTCCAACGTCAAAGGGCGAAAAACCGTCTATCAGGGCGATGGCCCACTACGTGAACCATCACCCTAATCAAGT
TTTTTGGGGTCGAGGTGCCGTAAAGCACTAAATCGGAACCCTAAAGGGAGCCCCCGATTTAGAGCTTGACGGGGAAAGCC
GGCGAACGTGGCGAGAAAGGAAGGGAAGAAAGCGAAAGGAGCGGGCGCTAGGGCGCTGGCAAGTGTAGCGGTCACGC
TGCGCGTAACCACCACACCCGCCGCGCTTAATGCGCCGCTACAGGGCGCGTCAGGTGGCACTTTTCGGGGAAATGTGCGC
GGAACCCCTATTTGTTTATTTTTCTAAATACATTCAAATATGTATCCGCTCATGAGACAATAACCCTGATAAATGCTTCAATA
ATATTGAAAAAGGAAGAGTCCTGAGGCGGAAAGAACCAGCTGTGGAATGTGTGTCAGTTAGGGTGTGGAAAGTCCCCAG
GCTCCCCAGCAGGCAGAAGTATGCAAAGCATGCATCTCAATTAGTCAGCAACCAGGTGTGGAAAGTCCCCAGGCTCCCCA
GCAGGCAGAAGTATGCAAAGCATGCATCTCAATTAGTCAGCAACCATAGTCCCGCCCCTAACTCCGCCCATCCCGCCCCTA
ACTCCGCCCAGTTCCGCCCATTCTCCGCCCCATGGCTGACTAATTTTTTTTATTTATGCAGAGGCCGAGGCCGCCTCGGCCTC
TGAGCTATTCCAGAAGTAGTGAGGAGGCTTTTTTGGAGGCCTAGGCTTTTGCAAAGATCGATCAAGAGACAGGATGAGG
ATCGTTTCGCATGATTGAACAAGATGGATTGCACGCAGGTTCTCCGGCCGCTTGGGTGGAGAGGCTATTCGGCTATGACT
GGGCACAACAGACAATCGGCTGCTCTGATGCCGCCGTGTTCCGGCTGTCAGCGCAGGGGCGCCCGGTTCTTTTTGTCAAGA
CCGACCTGTCCGGTGCCCTGAATGAACTGCAAGACGAGGCAGCGCGGCTATCGTGGCTGGCCACGACGGGCGTTCCTTGC
GCAGCTGTGCTCGACGTTGTCACTGAAGCGGGAAGGGACTGGCTGCTATTGGGCGAAGTGCCGGGGCAGGATCTCCTGTC
ATCTCACCTTGCTCCTGCCGAGAAAGTATCCATCATGGCTGATGCAATGCGGCGGCTGCATACGCTTGATCCGGCTACCTGC
CCATTCGACCACCAAGCGAAACATCGCATCGAGCGAGCACGTACTCGGATGGAAGCCGGTCTTGTCGATCAGGATGATCT
GGACGAAGAGCATCAGGGGCTCGCGCCAGCCGAACTGTTCGCCAGGCTCAAGGCGAGCATGCCCGACGGCGAGGATCTC
GTCGTGACCCATGGCGATGCCTGCTTGCCGAATATCATGGTGGAAAATGGCCGCTTTTCTGGATTCATCGACTGTGGCCGG
CTGGGTGTGGCGGACCGCTATCAGGACATAGCGTTGGCTACCCGTGATATTGCTGAAGAGCTTGGCGGCGAATGGGCTGA
CCGCTTCCTCGTGCTTTACGGTATCGCCGCTCCCGATTCGCAGCGCATCGCCTTCTATCGCCTTCTTGACGAGTTCTTCTGAG
CGGGACTCTGGGGTTCGAAATGACCGACCAAGCGACGCCCAACCTGCCATCACGAGATTTCGATTCCACCGCCGCCTTCTA
TGAAAGGTTGGGCTTCGGAATCGTTTTCCGGGACGCCGGCTGGATGATCCTCCAGCGCGGGGATCTCATGCTGGAGTTCTT
CGCCCACCCTAGGGGGAGGCTAACTGAAACACGGAAGGAGACAATACCGGAAGGAACCCGCGCTATGACGGCAATAAA
AAGACAGAATAAAACGCACGGTGTTGGGTCGTTTGTTCATAAACGCGGGGTTCGGTCCCAGGGCTGGCACTCTGTCGATA
CCCCACCGAGACCCCATTGGGGCCAATACGCCCGCGTTTCTTCCTTTTCCCCACCCCACCCCCCAAGTTCGGGTGAAGGCCC**

AGGGCTCGCAGCCAACGTCGGGGCGGCAGGCCCTGCCATAGCCTCAGGTTACTCATATATACTTTAGATTGATTTAAAACT
TCATTTTTAATTTAAAAGGATCTAGGTGAAGATCCTTTTTGATAATCTCATGACCAAAATCCCTTAACGTGAGTTTTCGTTCC
ACTGAGCGTCAGACCCCGTAGAAAAGATCAAAGGATCTTCTTGAGATCCTTTTTTTCTGCGCGTAATCTGCTGCTTGCAAAC
AAAAAAACCACCGCTACCAGCGGTGGTTTGTTTGCCGGATCAAGAGCTACCAACTCTTTTTCCGAAGGTAACTGGCTTCAG
CAGAGCGCAGATACCAAATACTGTCCTTCTAGTGTAGCCGTAGTTAGGCCACCACTTCAAGAACTCTGTAGCACCGCCTAC
ATACCTCGCTCTGCTAATCCTGTTACCAGTGGCTGCTGCCAGTGGCGATAAGTCGTGTCTTACCGGGTTGGACTCAAGACG
ATAGTTACCGGATAAGGCGCAGCGGTCGGGCTGAACGGGGGGTTCGTGCACACAGCCCAGCTTGGAGCGAACGACCTAC
ACCGAACTGAGATACCTACAGCGTGAGCTATGAGAAAGCGCCACGCTTCCCGAAGGGAGAAAGGCGGACAGGTATCCGG
TAAGCGGCAGGGTCGGAACAGGAGAGCGCACGAGGGAGCTTCCAGGGGGAAACGCCTGGTATCTTTATAGTCCTGTCGG
GTTTCGCCACCTCTGACTTGAGCGTCGATTTTTGTGATGCTCGTCAGGGGGGCGGAGCCTATGGAAAAACGCCAGCAACG
CGGCCTTTTTACGGTTCCTGGCCTTTTGCTGGCCTTTTGCTCACATGTTCTTTCCTGCGTTATCCCCTGATTCTGTGGATAACC
GTATTACCGCCATGCAT

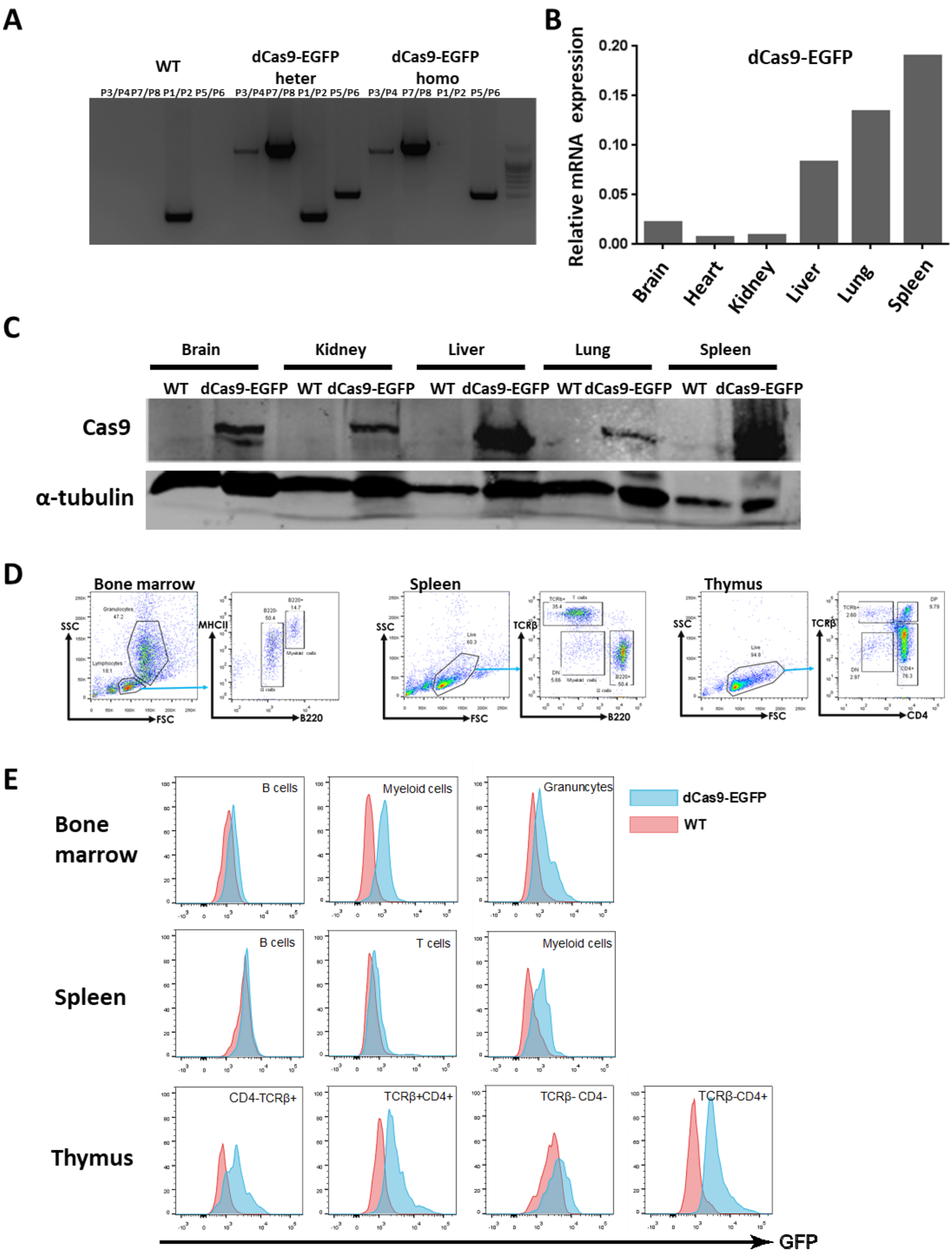
A
WT
dCas9-EGFP heter
dCas9-EGFP homo
P3/P4 P7/P8 P1/P2 P5/P6
B
dCas9-EGFP
Relative mRNA expression
Brain
Heart
Kidney
Liver
Lung
Spleen
C
Brain
Kidney
Liver
Lung
Spleen
WT
dCas9-EGFP
Cas9
α-tubulin
D
Bone marrow
Spleen
Thymus
SSC
FSC
MHCII
B220
TCRβ
CD4
E
Bone marrow
Spleen
Thymus
B cells
Myeloid cells
Granuncytes
T cells
CD4-TCRβ+
TCRβ+CD4+
TCRβ- CD4-
TCRβ-CD4+
dCas9-EGFP
WT
GFP

Figure S1

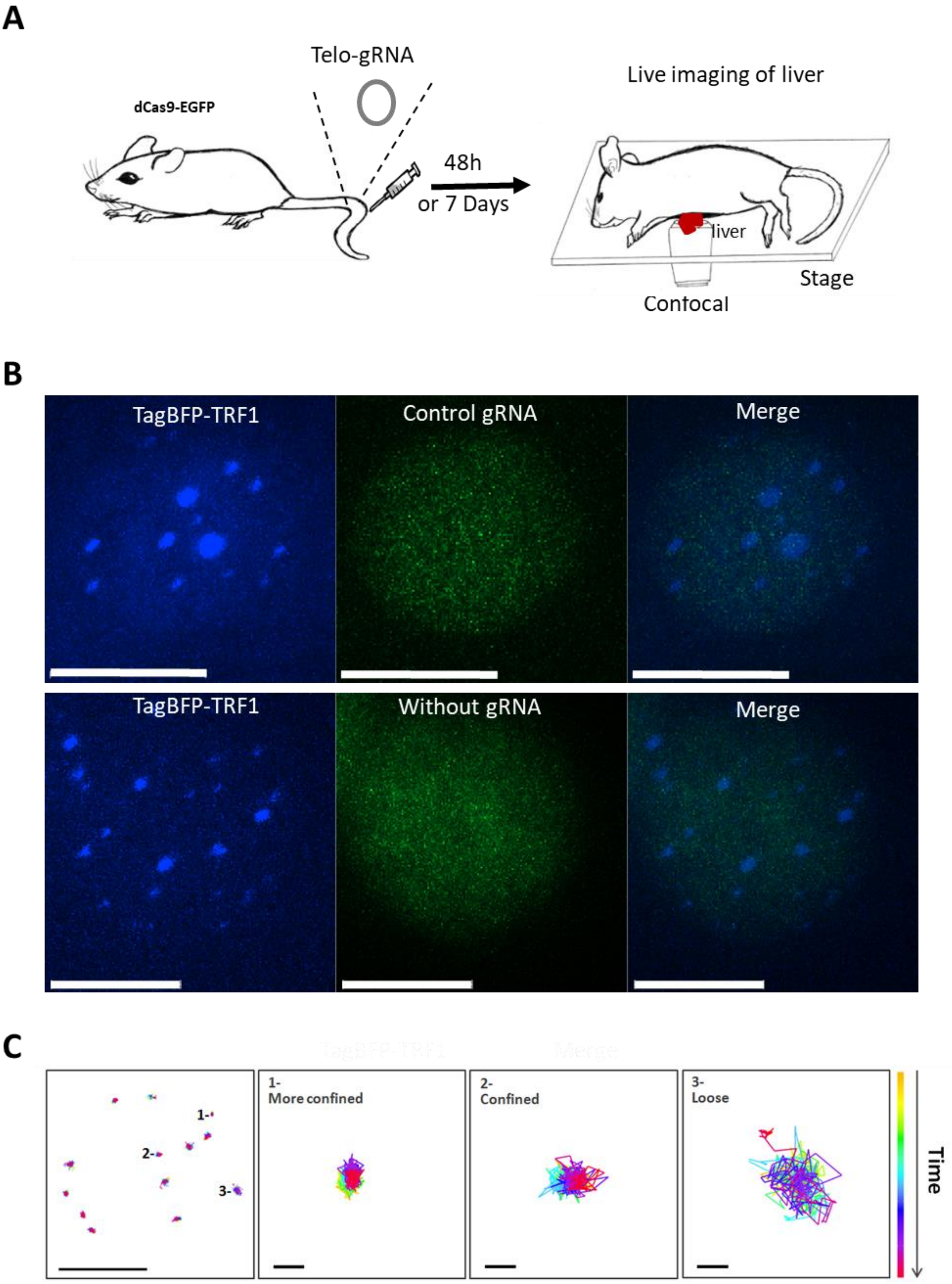
A
Telo-gRNA
dCas9-EGFP
48h
or 7 Days
Live imaging of liver
liver
Stage
Confocal
B
TagBFP-TRF1
Control gRNA
Merge
TagBFP-TRF1
Without gRNA
Merge
C
1-
2-
3-
1-
More confined
2-
Confined
3-
Loose
Time

Figure S2

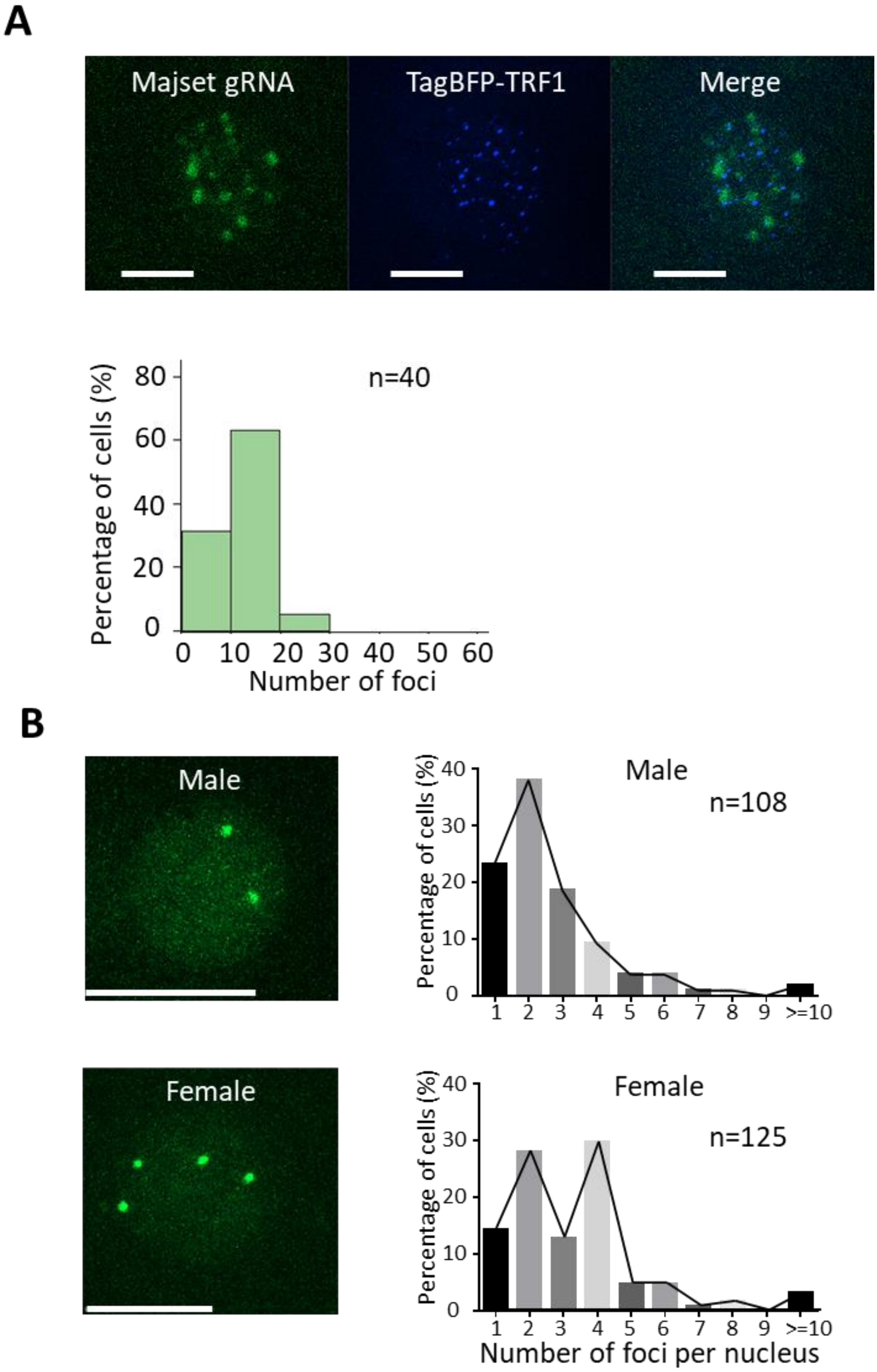
A
Majset gRNA
TagBFP-TRF1
Merge
Percentage of cells (%)
n=40
Number of foci
B
Male
n=108
Female
n=125
Percentage of cells (%)
Number of foci per nucleus

Figure S3

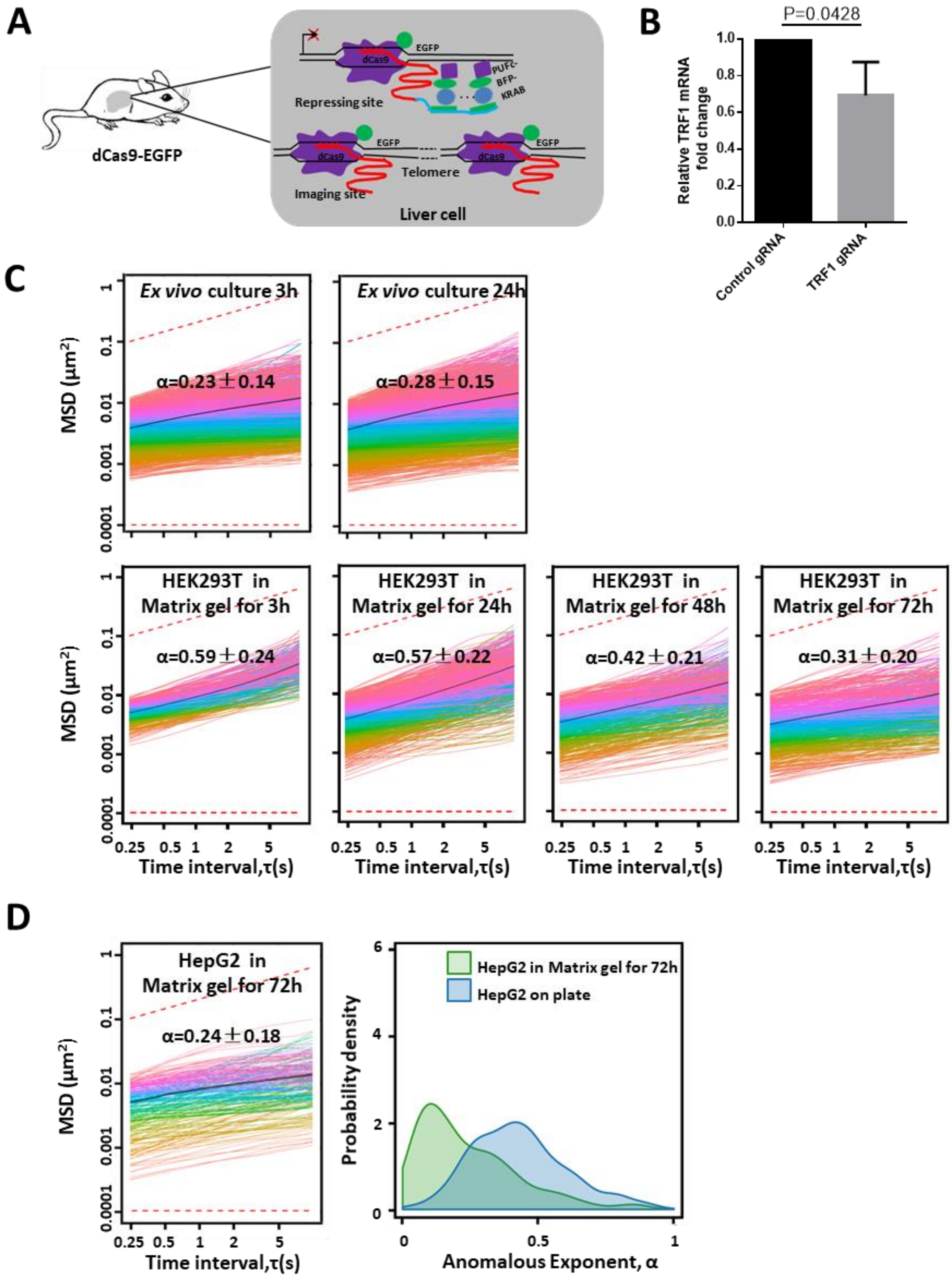
A
dCas9-EGFP
Repressing site
Imaging site
Telomere
Liver cell
B
P=0.0428
Relative TRF1 mRNA fold change
Control gRNA
TRF1 gRNA
C
Ex vivo culture 3h
α=0.23±0.14
Ex vivo culture 24h
α=0.28±0.15
HEK293T in Matrix gel for 3h
α=0.59±0.24
HEK293T in Matrix gel for 24h
α=0.57±0.22
HEK293T in Matrix gel for 48h
α=0.42±0.21
HEK293T in Matrix gel for 72h
α=0.31±0.20
MSD (μm²)
Time interval,τ(s)
D
HepG2 in Matrix gel for 72h
α=0.24±0.18
HepG2 in Matrix gel for 72h
HepG2 on plate
Probability density
Anomalous Exponent, α

Figure S4

E

	Number of mice	Experimental repeats	Cell number	Foci number	$MSD(t) = 4D_\alpha t^\alpha$		$MSD(t) = A(1-e^{-t/\delta}) + 4D_{macro}$		
					α	D_α ($\mu m^2 s^{-\alpha} x10^{-4}$)	D_{micro} ($10^{-3} \mu m^2/s$)	D_{macro} ($10^{-5} \mu m^2/s$)	$L_{confinement}$ (nm)
dCas9-EGFP mouse liver	5		129	1055	0.18 ± 0.15	29.49 ± 15.55	9.66 ± 4.53	9.07 ± 37.51	83.17 ± 36.01
Ex vivo culture 3h	4		84	1127	0.23 ± 0.14	16.01 ± 12.70	5.12 ± 3.75	10.09 ± 20.49	57.18 ± 28.44
Ex vivo culture 24h	4		87	1502	0.28 ± 0.15	17.22 ± 14.80	4.16 ± 3.42	11.35 ± 33.01	61.17 ± 38.22
HEK293T		3	67	590	0.46 ± 0.20	24.70 ± 15.59	5.50 ± 4.18	36.76 ± 54.38	76.72 ± 41.37
Hep1-6		3	64	867	0.52 ± 0.25	22.71 ± 14.42	4.95 ± 3.30	42.37 ± 56.31	74.77 ± 43.65
HepG2		3	58	691	0.45 ± 0.22	26.98 ± 15.21	4.98 ± 2.94	37.11 ± 73.95	86.10 ± 51.30
HEK293T in Matrix gel for 3h		2	30	295	0.59 ± 0.24	20.70 ± 9.33	5.48 ± 3.68	52.46 ± 39.68	69.33 ± 24.50
HEK293T in Matrix gel for 24h		4	74	827	0.57 ± 0.22	18.85 ± 12.24	3.64 ± 3.14	43.40 ± 55.07	68.48 ± 39.53
HEK293T in Matrix gel for 48h		4	42	485	0.42 ± 0.21	14.20 ± 9.59	3.62 ± 3.38	19.10 ± 25.81	57.10 ± 26.66
HEK293T in Matrix gel for 72h		4	53	597	0.31 ± 0.20	12.24 ± 10.98	4.68 ± 4.42	10.45 ± 23.93	49.72 ± 29.09
HepG2 in Matrix gel for 72h		3	26	243	0.24 ± 0.18	19.70 ± 14.90	7.30 ± 5.38	10.32 ± 17.02	62.52 ± 27.79

Figure S4 (continued)
